# Supplementary material for: Multidisciplinary interventions for reducing the avoidable displacement from home of frail older people: a systematic review
Source: BMJ Open. 2019 Nov 2;9(11):e030687. doi: 10.1136/bmjopen-2019-030687 (PMC6830674; doi:10.1136/bmjopen-2019-030687)
Supplement: Supplementary data [file bmjopen-2019-030687supp001.pdf]

APPENDIX 1: SEARCH STRATEGY

“home” OR “home hospital” OR “out-patient facility” OR “hospital in home” OR “Home care” OR “Domiciliary care” OR “outreach programme” OR “hospital outreach” OR “comprehensive elderly care” OR “home hospitalisation" AND “Frail elderly” OR “Elderly, Frail” OR “Functionally-impaired Elderly” OR “Aged OR elderly” OR “Aged, 80 and over” OR “Centenarians” OR “Nonagenarians” OR “Octogenarians" AND “Involuntary hospitalisation” OR “Avoidable hospitalisation" OR “Patient admission” OR “Voluntary Admission” OR “Patient Readmission” OR “30 Day Readmission” OR “Thirty Day Readmission” OR “Hospital Readmissions” OR “Readmissions, Hospital".

APPENDIX 2: DATA EXTRACTION FORM

Title
Author
Year
Methods
Participants
Patients
Classification of intervention
Intervention
Team
Person centeredness
Sustainability
Preventive aspect
Outcomes
Results
Conclusion

APPENDIX 3: RISK BIAS

Author	Random sequence generation (selection bias)	Allocation concealment (selection bias)	Blinding of participant and personnel (performance bias)	Blinding of outcome assessment (detection bias)	Incomplete outcome data (attrition bias)	Selective reporting bias
**Caplan et al**	Unclear risk (block randomisation)	Low risk (opaque envelopes)	Higk risk ("almost half conducted by a research assistant, with the rest performed by different members of the multidisciplinary team")	Low risk. Baseline characteristics are similar	"Unclear (10.0% of intervention participants and 13.0% of control participants withdrew from the study. The authors did not report reasons for withdrawal.)"	"High risk (Only data at 1 and 18 months were reported, yet measurements were taken at 1, 3, 6, 12 and 18 months)"
**Courtney et al**	Low risk (computer-generated)	High risk (not mentioned)	Unclear risk (not mentioned)	Low risk. Baseline characteristics are similar	High risk (attrition and 28% lack of compliance)	Low risk. Main outcomes are reported
**de Stampa el al**	High risk. 'Patients were recruited consecutively until the desired sample sizes were achieved.'		Low risk. Interventions occurs in different geographical areas	Low risk. Similar baseline characteristics	Low risk. Attrition is similar in both groups (deceased or institutionalised)	Low risk. Results are reported as stated in protocol
**Espinel-Bermudez et al**	High risk. Unclear criteria of selection (mentions quota and convenience sampling)		Low risk. (Two different hospitals, doing ordinary activities)	High risk. Baseline 'Length of stay' and comorbidity index differs between groups.	High risk. Unbalanced groups leads to different attrition loss.	Unclear
**Harris et al**	Low risk (computer-generated)	Unclear. Do not mention allocation process	Low risk (“Interviews by trained researchers who were not involved in patient care, but who were	Low risk (baseline results similar)	Unclear. Loss to follow-up: hospital at home: 14/143; hospital: 17/142	High risk (no mention to registered protocol)

			not blinded as to the intervention group")			
**Hendrix et al**	DNA	DNA	DNA	DNA	Low risk	High risk (absence of statistical analysis)
**Leung et al**	DNA	DNA	DNA	DNA	Low risk (no loss of patients)	High risk (statistical analysis incomplete)
**Low et al**	DNA	DNA	DNA	DNA	Low risk. Loss of 3 out of 262 patients	High risk. Does not control for covariables
**Meret-Hanke**	Low risk. "First, propensity score matching assumes that outcomes are independent of treatment assignment given an observable set of covariates (Dehejia & Wahba, 2002; Rosenbaum & Rubin, 1983). Second, the propensity score distributions of the treatment and comparison groups are assumed to overlap (Rosenbaum & Rubin, 1983). To satisfy these assumptions, we included covariates that strongly affect both the treatment decision and the outcome to effectively reduce bias (Smith & Todd, 2005)."		Low risk. Retrospective analysis	High risk. "For instance, individuals who enroll in PACE could be more averse to using hospitals than other individuals." "In addition, although we were able to match on most individual characteristics, we were unable to match on market, geographic, and historical factors."	Low risk. Different attrition bias analysis performed (baseline characteristics lost to attrition, effect of cases on outcomes)	Unclear

**Ong et al**	DNA	DNA	DNA	DNA	Low risk. 12% of deaths	Low risk. Main outcomes are reported
**Robinson et al**	Low risk. Threshold for RD is the minimum required for treatment ('risks core where the patients begin to receive the intervention(20% risk)')		Low risk. Quasi-experimental design (RD and ITS)	Low risk. Quasi-experimental design (RD and ITS)	Low risk. 'From this target group we identified eligible ‘High Risk’ patients using a predictive risk model (PRM) that could be run early in the patient's admission'	Low risk (“We measured whether the intervention was delivered according to the initial plan. In particular we measured whether patients received the discharge pharmacy and the nurse telephone follow-up services using program administrative records”)
**Rytter et al**	Low risk (computer generated)	Low risk (opaque envelopes)	High risk (not blinded to GP and nurses)	Low risk (baseline characteristics similar)	Unclear risk (Dropouts of lower functional ability patients)	Low risk. Main outcomes reported.

**Sahota et al**	Low risk (computer-generated)	Low risk (The randomisations will be requested through a PC with internet explorer and internet access. The system is located on a dedicated secure server within the University of Nottingham)	Low risk. Protocol: The RA collecting data and the research team analysing the data will be blinded to treatment allocation. The participant and ward staff will not be blinded to treatment allocation as the treating therapists will be liaising closely with ward staff to ensure optimal patient care. The three-month follow-up data will be collected by an RA blinded to treatment allocation.	Low risk (The groups appeared well balanced at baseline)	Low risk (212 participant, 106 in each arm)	Low risk (similar outcomes as protocol)
**Stranges et al**	DNA	DNA	DNA	Low risk (Propensity Score Matching)	Low risk (retrospective analysis)	Low risk. Main outcomes are reported
**Young et al**	High risk. Patients were recruited consecutively		Unclear. Evaluation occurs within policy change	Low risk. Baseline characteristics are similar	Unclear. Mortality rate is similar after 1 year	Low risk. Main outcomes are reported

DNA: Do not apply

APPENDIX 4: PRISMA CHECKLIST

Section/topic	#	Checklist item	Reported on page #
TITLE			
Title	1	Identify the report as a systematic review, meta-analysis, or both.	1
ABSTRACT			
Structured summary	2	Provide a structured summary including, as applicable: background; objectives; data sources; study eligibility criteria, participants, and interventions; study appraisal and synthesis methods; results; limitations; conclusions and implications of key findings; systematic review registration number.	2
INTRODUCTION			
Rationale	3	Describe the rationale for the review in the context of what is already known.	3
Objectives	4	Provide an explicit statement of questions being addressed with reference to participants, interventions, comparisons, outcomes, and study design (PICOS).	4
METHODS			
Protocol and registration	5	Indicate if a review protocol exists, if and where it can be accessed (e.g., Web address), and, if available, provide registration information including registration number.	4
Eligibility criteria	6	Specify study characteristics (e.g., PICOS, length of follow-up) and report characteristics (e.g., years considered, language, publication status) used as criteria for eligibility, giving rationale.	4-5
Information sources	7	Describe all information sources (e.g., databases with dates of coverage, contact with study authors to identify additional studies) in the search and date last searched.	4
Search	8	Present full electronic search strategy for at least one database, including any limits used, such that it could be repeated.	4 AND APPENDIX
Study selection	9	State the process for selecting studies (i.e., screening, eligibility, included in systematic review, and, if applicable, included in the meta-analysis).	5
Data collection process	10	Describe method of data extraction from reports (e.g., piloted forms, independently, in duplicate) and any processes for obtaining and confirming data from investigators.	5
Data items	11	List and define all variables for which data were sought (e.g., PICOS, funding sources) and any assumptions and simplifications made.	4
Risk of bias in individual studies	12	Describe methods used for assessing risk of bias of individual studies (including specification of whether this was done at the study or outcome level), and how this information is to be used in any data synthesis.	5
Summary measures	13	State the principal summary measures (e.g., risk ratio, difference in means).	DNA
Synthesis of results	14	Describe the methods of handling data and combining results of studies, if done, including measures of consistency (e.g., I^2) for each meta-analysis.	DNA

Risk of bias across studies	15	Specify any assessment of risk of bias that may affect the cumulative evidence (e.g., publication bias, selective reporting within studies).	5 and APPENDIX
Additional analyses	16	Describe methods of additional analyses (e.g., sensitivity or subgroup analyses, meta-regression), if done, indicating which were pre-specified.	DNA
RESULTS			
Study selection	17	5-6	4
Study characteristics	18	For each study, present characteristics for which data were extracted (e.g., study size, PICOS, follow-up period) and provide the citations.	6-11
Risk of bias within studies	19	Present data on risk of bias of each study and, if available, any outcome level assessment (see item 12).	12 and APPENDIX
Results of individual studies	20	For all outcomes considered (benefits or harms), present, for each study: (a) simple summary data for each intervention group (b) effect estimates and confidence intervals, ideally with a forest plot.	12-14
Synthesis of results	21	Present results of each meta-analysis done, including confidence intervals and measures of consistency.	DNA
Risk of bias across studies	22	Present results of any assessment of risk of bias across studies (see Item 15).	DNA
Additional analysis	23	Give results of additional analyses, if done (e.g., sensitivity or subgroup analyses, meta-regression [see Item 16]).	DNA
DISCUSSION			
Summary of evidence	24	Summarize the main findings including the strength of evidence for each main outcome; consider their relevance to key groups (e.g., healthcare providers, users, and policy makers).	6-11
Limitations	25	Discuss limitations at study and outcome level (e.g., risk of bias), and at review-level (e.g., incomplete retrieval of identified research, reporting bias).	16
Conclusions	26	Provide a general interpretation of the results in the context of other evidence, and implications for future research.	15-17
FUNDING			
Funding	27	Describe sources of funding for the systematic review and other support (e.g., supply of data); role of funders for the systematic review.	17

From: Moher D, Liberati A, Tetzlaff J, Altman DG, The PRISMA Group (2009). Preferred Reporting Items for Systematic Reviews and Meta-Analyses: The PRISMA Statement. PLoS Med 6(7): e1000097. doi:10.1371/journal.pmed1000097
