# Supplementary material for: Multidisciplinary interventions for reducing the avoidable displacement from home of frail older people: a systematic review
Source: BMJ Open. 2019 Nov 2;9(11):e030687. doi: 10.1136/bmjopen-2019-030687 (PMC6830674; doi:10.1136/bmjopen-2019-030687)
Supplement: Author's manuscript [file bmjopen-2019-030687.draft_revisions.pdf]

BMJ Open is committed to open peer review. As part of this commitment we make the peer review history of every article we publish publicly available.

When an article is published we post the peer reviewers' comments and the authors' responses online. We also post the versions of the paper that were used during peer review. These are the versions that the peer review comments apply to.

The versions of the paper that follow are the versions that were submitted during the peer review process. They are not the versions of record or the final published versions. They should not be cited or distributed as the published version of this manuscript.

BMJ Open is an open access journal and the full, final, typeset and author-corrected version of record of the manuscript is available on our site with no access controls, subscription charges or pay-per-view fees (http://bmjopen.bmj.com).

If you have any questions on BMJ Open's open peer review process please email info.bmjopen@bmj.com

BMJ Open

Multidisciplinary interventions for reducing the avoidable displacement from home of frail older people: a systematic review

Journal:	*BMJ Open*
Manuscript ID	bmjopen-2019-030687
Article Type:	Research
Date Submitted by the Author:	27-Mar-2019
Complete List of Authors:	Sempé, Lucas; University of East Anglia, School of International Development<br>Billings, Jenny; University of Kent, Centre for Integrated Care Research<br>Lloyd-Sherlock, Peter; University of East Anglia, School of International Development
Keywords:	healthcare, older people, social care, systematic review, avoidable displacement from home

SCHOLARONE™
Manuscripts

Multidisciplinary interventions for reducing the avoidable displacement from home of frail older people: a systematic review

Lucas Sempé,[1] Jenny R. Billings,[2] Peter Lloyd-Sherlock[1]

[1] School of International Development, University of East Anglia, Norwich, NR4 7TJ, UK

[2] Centre for Health Services Studies, University of Kent, Canterbury, CT2 7NZ, UK

Corresponding author: Lucas Sempe. E-mail l.sempe@uea.ac.uk / Telephone: +447907994151

Word count: 3619

For peer review only

For peer review only - http://bmjopen.bmj.com/site/about/guidelines.xhtml

ABSTRACT

Objectives: To synthesise existing literature on interventions addressing ADH for older people with multimorbidity or frailty.

Design: A systematic search of the main bibliographic databases was conducted to identify studies relating to interventions addressing ADH for older people. Studies focusing on one specific condition or interventions without multidisciplinary teams were excluded. A narrative synthesis of data was conducted, and themes were identified by using an adapted thematic framework analysis approach.

Results: Overall 3927 articles were identified and were 364 were retained for full text screening. Fifteen studies were included in the narrative review. Four themes were identified: (1) types of interventions; (2) composition of teams; (3) intervention effectiveness; and (4) outcomes measurement.

Conclusions: The review identified some potential interventions and relevant topics to be addressed in order to develop effective and sustainable interventions to reduce the ADH of older people. However the review was not able to identify robust impact evidence either in terms of quantity or quality from the studies presented, such that the strength of the evidence cannot be considered sufficiently strong to inform policy or interventions seeking to reduce ADH. This finding reflects the complexity of these interventions and a lack of systematic data collection.

Keywords: avoidable displacement from home, healthcare, older people, social care, systematic review

STRENGTHS AND LIMITATIONS OF THIS STUDY

- This is a timely systematic review of interventions involving integrated care for older people, and these are situated within a new policy framework.
- This review examines interventions addressing a significant public health and social care challenge in the future.

For peer review only - http://bmjopen.bmj.com/site/about/guidelines.xhtml

- Evidence in this area is predominately from quasi-experiments and observational studies with an important number of studies presenting unclear results and tion effectiveness on hospital and emergency department admissions and readmissions.

INTRODUCTION

Rationale

Between 2017 and 2050 the global population of people aged 60 years or over is expected to more than double, reaching 2.1 billion people. In 2015 around two thirds of this population lived in low or middle-income countries, rising to 80% by 2050 [1]. This demographic transition creates challenges for both social services and health national systems. This includes rising numbers of hospitalisations, many of which are potentially avoidable [2,3]. Responses to this challenge include an increased emphasis on interventions that integrate different forms of health and social care provision, working across different care settings[4]. Interventions to promote and support integrated care are now widely advocated at many policy levels as an approach to deal with varying and complex health needs of older people. However, the effectiveness of integrated care approaches remains inconsistent across care settings and different interventions [5].

Given the wide diversity that integrated care interventions can take and their complex interactions with other parts of health and social care systems, it is important to establish clear parameters about where they are situated in relation to different care settings and to clarify outcomes of interest. The framework of ‘avoidable displacement from home’ (ADH) offers a comprehensive approach to achieve this, by encompassing a number of effects: hospital (re)admissions that are amenable to prevention or which are suited to outpatient care, excessive hospital stays and unnecessary admissions into residential care homes. ADH incorporates interventions that emphasise three simultaneous characteristics which are considered relevant in terms of efficacy and dignity: person-centredness [6], place-based care [7] and sustainability [8].

Objective

Our specific objective was to conduct a systematic review to identify interventions addressing displacement from home of older people with multimorbidity or frailty. The review focused on studies with the following characteristics: home-based interventions by any type of

For peer review only - http://bmjopen.bmj.com/site/about/guidelines.xhtml

multidisciplinary team that aimed at reducing ADH in older patients with multimorbidity or frailty. A second objective was to characterise those interventions in order to inform policy.

METHODS

Protocol and registration

The protocol for the systematic review was listed on the PROSPERO prospective register on 03 September 2018. The registration number is CRD42018108116 (supplementary material).

Eligibility criteria

Following the review objectives, any type of quantitative study (RCTs, quasi-experiments and observational data) that fitted our criteria was included in the review process. The eligibility criteria guiding the search selected publications specifically focusing on older people (defined as aged over 60 years) who received any kind of home-based intervention by a multidisciplinary team. In order to assess any effect on ADH, the studies needed to record at least one of the following outcomes: hospital length of stay, hospital or emergency department admissions or readmissions.

Information sources and searches

This is a systematic review of articles indexed in the following electronic databases: the American National Library of Medicine and the National Institutes of Health (PubMed), Scopus, Cochrane Library (Central and CDRS), CINAHL, Social Care Online, Web of Science as well as the database of the Latin American and Caribbean Health Sciences Literature (LILACS). We also examined the reference lists of the selected studies.

The search was conducted without any language restriction and focused on articles published after the year 2000. The review only included articles published in Portuguese, English, and Spanish, because of our familiarity with these languages. The databases were consulted in September 2018 and the articles were moved into Mendeley 1.18. The duplicates have been deleted.

Search strategy

An initial scoping of the literature was conducted at inception of the study and those findings were used to define the search strategy. In order to take an inclusive approach we included a variety of key search terms linking three different topics, namely: ‘home-based care’, terms to identify ‘older adults’ in conjunction with ‘avoidable hospitalisations’ utilising Medical Subject Headings (MeSH). The search syntax is provided in Appendix 1.

For peer review only - http://bmjopen.bmj.com/site/about/guidelines.xhtml

Study selection and data extraction process

The data extraction process had two stages. Two authors (LS, JB) independently screened all titles and abstracts identified from searches to determine which met the inclusion criteria. They also independently screened full-text articles for inclusion or exclusion, with discrepancies resolved by discussion and by consulting a third author (PLS). All potentially relevant papers excluded from the review at this stage are listed as excluded studies, with reasons provided in the Characteristics of Excluded Studies table. A data extraction form was developed to extract and appraise each study according to the aims of the study.

Assessment of risk of bias of individual studies

Risk of bias was assessed using the Cochrane Collaboration tool [9] and was rated as low, unclear, or high. Ratings of low risk of bias indicate high confidence in study findings. Ratings of unclear risk of bias indicate the presence of probable bias or flaws that raise reservations about study findings. High risk of bias are indicators of poor study quality, and results are considered with caution. One author (LS) assessed the risk of bias of the included studies, which was reviewed by both additional authors (JB, PLS). Any disagreements were resolved by discussion to reach consensus.

Synthesis of results

Thematic analysis was then conducted within and across categories to identify key themes. All authors deliberated and agreed on these themes as well as their validity and implications. Since one objective of this review is to inform policy, descriptive information about all interventions is presented and discussed in the results section, although only those presenting low or unclear risk of bias are summarised and discussed in terms of their effectiveness.

Patient and public involvement

Patients and the public were not involved in the design or analysis of this review.

RESULTS

Study selection

A total of 3927 articles were identified. After screening titles and abstracts, 364 full-texts were reviewed, of which fifteen articles met the inclusion criteria (Figure 1).

For peer review only

(Insert Figure 1)

Study characteristics

The systematic review identified 15 interventions, including four different types: transitional care (n=8), case-management services (n=3), hospital-at-home (n=3), and a medication-review intervention (n=1). Five studies were RCTs, eight were quasi-experiments (four prospective controlled pre-post design and one retrospective controlled pre-post design) and three were retrospective non-controlled observational studies. Four interventions took place in the United States [10–13], while two interventions were set in the United Kingdom [14,15], New Zealand [16,17], and Singapore [18,19]. Additionally, there was one intervention each in France [20], Australia [21], Denmark [22], Mexico [23] and Hong Kong [24]. See Table 1 for a summary of study characteristics.

For peer review only

For peer review only - http://bmjopen.bmj.com/site/about/guidelines.xhtml

Table 1. Summary of studies

Study	*Year*	*Methods*	*Number of participants*	*Demographics*	*Classification of intervention*	*Intervention*	*Team*	*Outcomes*	*Quality and bias assessment*
Caplan et al	2004	RCT	739	>75	Case-management	Comprehensive geriatric assessment and multidisciplinary intervention on older patients sent home from the emergency department (ED). A nurse formulates a care plan, initiates urgent interventions and referrals, and presents the patient's history at a weekly interdisciplinary team meeting at which further interventions or referrals can be ordered.	Meetings attended by a geriatrician or a geriatric registrar, nurses, physiotherapists, and occupational therapists	All hospital admissions within 30 days<br>Elective and emergency admissions (days to first emergency admission)<br>Nursing home admissions<br><br>Mortality<br><br>Physical function (Barthel Index)<br>Instrumental activities of daily living<br>Cognitive function	Unclear risk of bias
Courtney et al	2009	RCT and economic study (in additional paper)	128	>65	Transition care	Comprehensive nursing and physiotherapy assessment and individualised programme of exercise strategies. Home visit by a nurse. Telephone follow-up commencing in the hospital and continuing for 24 weeks after discharge.	Nurse and physiotherapist	Hospital readmissions and emergency GP visits	Unclear quality and risk of bias
de Stampa el al	2014	Prospective controlled	428	>64	Case-management	COPA. Multi-disciplinary comprehensive geriatric needs assessment. This	Case managers (geriatric nurses), primary care	Unplanned hospitalisation (via ED)<br>Any hospitalisation	Unclear risk of bias

For peer review only - http://bmjopen.bmj.com/site/about/guidelines.xhtml

		pre-post design				includes an individual care plan, care management programmes, evidence-based protocols, and regular reassessments of patient needs	physician and a psychologist	(1 year)<br><br>Instrumental Activity of Daily Living<br>Activity of Daily Living<br>Hierarchical<br>Cognitive Performance<br>Depression Rating<br>Aggressive Behaviour<br>Pain Scale	
Espinel-Bermudez et al	2011	prospective controlled pre-post design	70	>60	Hospital at home	Programme of domiciliary attention for chronically ill patients. Two educational sessions with patient carers. Home visits, provision of medical equipment and medication. Telephone line available for careers	Two teams. The first in based in the hospital and second makes home visits (doctor, nurse, psychologist and a medical resident).	Hospitals readmissions over one year (type, number of times, days to readmission, and length of stay)<br><br>Quality of life (Sickness impact profile)	High risk of bias
Harris et al	2005	RCT	285	>55	Hospital at home	Seven days per week/10 hour per day nursing availability. 24 hour on-call geriatrician. Patient-centred planning. Daily nursing review and adjustment of individual care plan. Intensive home support with up to 24 hours support from a live-in home care professional. Multidisciplinary team support. Rehabilitation in the patient's home. A discharge	Nursing-led. Occupational therapist, physiotherapist, and social workers	Readmissions to hospital (first readmission in days 1-10,11-30 and 31-90)<br><br>Physical and mental function (functional independence)<br>Instrumental activities of daily living<br>Mental status<br>Self-reported recovery<br>Health status (SF-36)<br>Falls<br>Satisfaction with	Low risk of bias

For peer review only - http://bmjopen.bmj.com/site/about/guidelines.xhtml

						hand-over to ongoing support services.		intervention<br><br>Costs	
Hendrix et al	2013	Prospective controlled pre-post design	47	>60	Transition care	Hospital visit before the patient's discharge to initiate a relationship with the patient and begin planning for home care. Home visits by team. Educational materials for caregivers and information on community-based resources.	Nurse practitioner, social worker, occupational therapist	Emergency department visits and rehospitalisation at 30 and 60 days after hospital discharge.	Low quality and high risk of bias
Leung et al	2015	Matched-control quasi-experimental study	78	T: 80.2±7.7<br>C: 80.5±8.5	Hospital at home	The virtual ward physician might conduct a physician home visit within the first week after the patient was discharged. Based on the health assessment results, the virtual ward nurses planned the schedule of home visits, which could range from daily to once every 2 weeks.	Nurses and physicians	Unplanned emergency hospital readmissions, quality of life, emergency attendances	Low quality and unclear risk of bias
Low et al	2015	Prospective controlled	259	>65	Transition care	An individualised patient-centred care plan drawn up for each patient after an	Multi-disciplinary team comprising of a family physician,	Emergency department attendances (after 3 and 6 months)	Low quality and high risk of bias

For peer review only - http://bmjopen.bmj.com/site/about/guidelines.xhtml

		pre-post design				initial home visit assessment. The frequency and timing of future home visits and the need to involve other members of the multi-disciplinary team is identified during the initial assessment and communicated to all team members. Nurses make telephone call reviews based on patients' care needs and are accessible to patients by phone during office hours.	a nurse case manager, a physiotherapist, an occupational therapist, a speech therapist and a medical social worker. Nurses function as case managers.	Admissions to all hospitals (after 3 and 6 months)<br><br>Length of stay (after 3 and 6 months)<br><br>Specialist outpatient clinic visits (after 3 and 6 months)	
Meret-Hanke	2011	Prospective controlled pre-post design	6992	>65	Case-management	PACE: Case-management services. Services provided in Day Health Centre (i.e.: recreational therapy nursing; Social services, including caregiver training, support groups, and respite care services)<br>Dentistry). Patients attend centres from rarely to 7 days a week, depending on their care plans.	Comprehensive range of health care professionals	Hospital Use (as average days per month in hospital) (each 6 months for up to two years)	High risk of bias
Ong et al	2017	Retrospective non-controlled observational	107	>51.9	Medication review	Home-based medication reviews	Pharmacist and care coordinator	Hospital admissions<br>ED visits<br>Length of stay (days)	High risk of bias

For peer review only - http://bmjopen.bmj.com/site/about/guidelines.xhtml

Robinson et al	2015	Retrospective controlled pre-post design	5239	>65	Transition care	Transition intervention: post-discharge component (telephone assessment, education, support by team) to identify problems not dealt with during discharge, assist with self-management, and ensure appropriate health and social support	A geriatrician, a pharmacist and cultural support workers	28 day readmission rates<br>ED visits	Low risk of bias
Rytter el al	2010	RCT	331	>78	Transition care	Home visit by a GP and a district nurse one week after discharge, followed by two contacts after three and eight weeks	GP and nurse	Readmission rate within 26 weeks after discharge<br><br>Control of medication, evaluated 12 weeks after discharge	Low risk of bias
Sahota et al	2017	RCT and economic study	250	>70	Transition care	Community In-Reach rehabilitation and Care Transition (CIRACT). A comprehensive assessment of the patient's ability to perform different tasks and creation of a rehabilitation plan. Form links with the appropriate community services to ensure a smooth and effective discharge. Following discharge, team home visits to assess the level of rehabilitation required. Further follow-up visits as deemed necessary	A senior occupational therapist (transition coach), a senior physiotherapist and an assistant practitioner, linked directly to a social services practitioner, working across multiple boundaries with patients and their carers.	Hospital length of stay<br><br>Readmission (28 and 91 days post-discharge)<br><br>Total time in bed days<br><br>Functional ability<br>Co-morbidity and health-related quality of life<br><br>Costs	Unclear risk of bias

For peer review only - http://bmjopen.bmj.com/site/about/guidelines.xhtml

						and appropriate referral to additional community services.			
Stranges et al	2015	Retrospective cohort study	1144	>60	Transition care	Phone call 2 to 4 days after discharge. The patient seen in clinic, ideally within 1 week of discharge, by a social worker and a health professional. Home visit by health professional, who conducts a modified geriatric assessment with a focus on the reason for hospital admission.	Geriatric physicians, nurse practitioners, clinical pharmacists and social workers	30-day readmission rates	Low risk of bias
Young et al	2005	Prospective controlled pre-post design	1648	>63	Transition care	An intermediate care (IC) service. Joint care management team (multi-disciplinary, multi-agency) assesses need and purchases services for individuals delivered through a primary care trust. Patients accepted for IC are then assessed by each discipline in the team and a care plan is developed to be delivered by the care assistants. Patients receive input for up to 6 weeks, according to need.	Nurses (5), occupational therapists (2), care assistants (15), a dietician (half time) and access to psychiatric nurses	Nottingham Extended Activities of Daily Living score<br>Barthel Index<br>Hospital Anxiety and Depression score<br><br>Mortality (3, 6 and 12 months)<br>Readmission to hospital (3, 6 and 12 months)<br>Readmission to hospital number of days (3, 6 and 12 months)<br>New institutional care placements (3, 6 and 12 months)	Unclear risk of bias

For peer review only - http://bmjopen.bmj.com/site/about/guidelines.xhtml

Risk of bias

Two RCT studies were considered as presenting low risk of bias [16,22], while the others present an unclear risk of bias due selective reporting that was discordant with the protocol [21], due to implementation problems and power analysis [14] and due to the randomisation process [11]. Two quasi-experimental studies present low risk of bias [13,17], while the others were assessed as showing unclear risks due to a lack of randomisation [11,20,23] and programme implementation issues [17]. The remaining studies presented a high risk of bias due to elements of the methodological design or analysis [12,15,18,19,24] and baseline differences [10,23]. The risk of bias of individual studies is detailed in Annex 3.

Type of outcomes

Ten studies reported on hospital readmissions [12–17,22–24], while six reported hospital admissions [10,14,18–21]. Only one study presented both outcomes [14]. Six studies reported emergency department admissions [11,17–21] and five measured length of hospital stay [10,14,18,19,23]. Additionally, three studies measured emergency GP visits [11,12,24], and one also measured control of medication [22].

Other outcomes, such as functional status and quality of life or depression, were measured in several prospective studies [14–16,20,23,24]. Mortality was assessed in two studies [15,21] and there was a costs analysis in three studies [11,14,16], one of them in a separate study [25]. Finally, patient satisfaction is assessed in one study [16].

Results were assessed over a wide range of time periods. One study started measuring results 10 days after the intervention started [16], while the most common follow-up period ranged from 1 to 6 months [10–17,19,21,22,24]. Two studies extended the follow up period to one year [15,23] and one measured the effects after two years [10].

Transitional care

Eight studies looked at transitional care interventions. Low et al [19] present a hospital-based intervention where patients received a comprehensive needs assessment in the home, followed by an individualised care plan that included medical and nursing care, patient education and coordination of care with other clinical specialists and community services. Nursing care included visits and follow-up phone calls. Robinson et al [17] describe an intervention that included an improved discharge process with two components: the identification of high risk patients and a post-discharge component that consisted of a nurse telephone assessment, along with education, and support by community nurses on the first

For peer review only - http://bmjopen.bmj.com/site/about/guidelines.xhtml

and third days post-discharge. Sahota et al [14] present the Community In-Reach rehabilitation and Care Transition (CIRACT) service. CIRACT developed a pre-discharge functionality assessment tool and a rehabilitation plan. After discharge, the service team performed home visits to assess progress and to provide appropriate referral to additional community services. Young et al [15] report on a city-wide intermediate care service where a joint care management team assessed patient needs and purchased support and rehabilitation from specialised teams. Courtney et al [11] study the Older Hospitalised Patients' Discharge Planning and In-home Follow-up Protocol (OHP-DP), developed by the authors, which started within 72 hours of hospital admission and continued for 6 months after transfer home. A nurse and a physiotherapist performed a comprehensive patient assessment and developed an individualised care plan in consultation with the patient, health professionals, family, and caregivers. The plan included exercises, muscle stretching and strengthening, balance training and walking. The intervention also has a weekly follow-up telephone call after discharge. Hendrix et al [12] present results of the Transitional Care (TLC) Partners Programme, which was led by a nurse practitioner and provided after-discharge medical care to patients for a time-limited period, with the addition of an occupational therapist and a social worker, who conducted separate home visits. Rytter et al [22] describe a joint home visit involving a GP and district nurse conducted after one week of discharge and two contacts with the GP in the third and eighth week after discharge, either in the GP's clinic or as a home visit depending on the patient's overall condition. Finally, Stranges et al [13] decribe a multidisciplinary team of geriatric physicians, nurse practitioners, clinical pharmacists, and a social worker who assisted patients transitioning to the community after unscheduled hospitalizations, long-term-care facility stays, or emergency department (ED) visits. The intervention began with a pharmacist's phone call 2 to 4 days after discharge, followed by a clinic appointment with a social worker and geriatrician or nurse within a week of discharge. Home visits and intensive follow-up for up to 3 months were provided as needed.

In summary, transitional or intermediate care interventions focus on the post-discharge component and cover a range of different actions such as the elaboration of individualised patient-centred care plans [11,19], telephone assessment and education [17] and home visits for short periods of time [13–15,19]. These may lead to referrals to other services such as rehabilitation or additional community services [14,15].

For peer review only - http://bmjopen.bmj.com/site/about/guidelines.xhtml

Case-management services

Three studies presented case-management services. Caplan et al [21] provide information on a comprehensive geriatric assessment performed in an emergency department, in addition to follow up visits by a hospital-based multidisciplinary outreach team over four weeks. Meret-Hanke [10] describes the Programme of All-Inclusive Care for the Elderly (PACE) , which provided comprehensive medical and social services to targeted community-dwelling older individuals. PACE organised their services in a Day Health Centre which members attended with varying frequency (from occasionally to daily), depending on their care plans. Both of these case-management interventions shared some characteristics: they included geriatric assessments, the elaboration of individualised care plans and coordination between care managers and/or multidisciplinary meetings where further interventions or referrals could be ordered [20,21].

Hospital-at-home interventions

Three studies present information regarding hospital-at-home interventions. Espinel et al [23] describe a programme with visits by a multidisciplinary team, the provision of educational sessions and a telephone line available to carers, in addition to the provision of medical equipment and medication. Harris et al [16] describe an intervention that supported 10 hours per day of nursing, 24-hour on-call geriatricians, daily patient-centred planning by nurses and, if needed, the upgrading of individual care plans to offer intensive home support with up to 24-hours of a live-in home care professional. Finally, Leung et al [24] present a virtual ward service, whereby a nurse and a GP separately visit the home in the first week after discharge. The nurse visited the home every two weeks, providing psychosocial support to both patients and their carers. Additionally, a hotline consultation service was put in place.

Medication-review intervention

Ong et al is the only study that focused on medication-review [18]. They present a home-based medication review programme led by a pharmacist and accompanied by a care coordinator, in liaison with other medical specialists. The program provided pharmacological counselling, and the identification of drug-related problems. The care coordinator focused on identifying social and environmental issues and developing appropriate interventions to overcome these barriers, which included the installation of grab bars at older people's homes to reduce risks of falls, the acquisition of mobility aids to improve home functioning, and the provision of complementary patient education and caregiving training.

For peer review only - http://bmjopen.bmj.com/site/about/guidelines.xhtml

Multidisciplinary team

A wide variety of multidisciplinary teams, in terms of composition and size, were found across the studies. Two interventions were pharmacist-led, accompanied by other staff such as care coordinators [18] or cultural workers in a context of diversity [17]. All other studies described nurses playing leading roles, such as case managers [16,19,20]. Geriatricians were present in only one study [21] while family or primary care doctors were part of the intervention in three studies [19,20,23]. Other health workers such as physiotherapists, occupational therapists, psychologists, speech therapists and dieticians were found in all studies [10,14,15,19–21,23]. Only three interventions included social workers [10,14,16].

Effects on hospital and ED admissions and readmissions

Three studies presenting low risk were assessed in terms of their intervention effectiveness on hospital and ED admissions and readmission. Only Stranges et al [13] find a significant reduction in 30 day readmission rates among matched compliers (n = 217, 11.7% vs 17.3%, treatment and control respectively; p <.001) and also a positive delay in terms of time to readmission, which was significantly longer among those receiving the intervention (18 ± 9 days compared with 12 ± 9 days with usual care; p = .015). Neither Harris et al [16] (n=208, 76.8% vs 69.2%, treatment and control respectively; p >.05) nor Robinson et al [17] (n=2486, Regression discontinuity parameter estimation= -1.60 [SD 3.10]; p >.05) find statistically significant differences in terms of readmissions.

Five studies presenting unclear risk were also assessed. Caplan et al [21] report a statistically significant reduction of hospital visits after 30 days (16.5% vs 22.2%, treatment and control respectively; p<.048), a lower rate of emergency admissions during the 18-month follow-up (44.4% vs 54.3%, treatment and control respectively; p<.007), and longer time to first emergency admission (382 vs 348 days, treatment and control respectively; p<.011). De Stampa et al [20] report a lower risk of having at least one unplanned hospital admission after covariable adjustment (OR=0.39; 95% CI=0.16–0.98), while planned admissions significantly increased in the intervention group (OR=3.59, 95% CI= 1.02–12.7). Finally, Rytter et al [22] report a reduction in readmissions twenty-six weeks after discharge (40% and 52%, treatment and control respectively; p=.03), with a relative risk reduction (RRR) of 23%. They also report a reduction in the number of days spent in hospital for the intervention group (p = 0.07, Wilcoxon rank-sum test) although their report is incomplete regarding statistical parameters.

For peer review only - http://bmjopen.bmj.com/site/about/guidelines.xhtml

The other remaining studies do not present significant or robust differences in outcomes [14,15].

Healthcare expenditure

Harris et al report the costs of hospital-at-home (NZ$6524) intervention were almost double standard hospital care (NZ$3525) [16], while Sahota et al found a net monetary benefit of £1,932 (95% CI= £2,134 to £5,863), although this has to be interpreted with caution given the wide confidence intervals [14]. Additionally, Courtey et al's intervention estimates an average cost saving of $333 for 24 weeks period per patient, and a net-monetary-benefit per individual of $7,907 [25].

DISCUSSION

Hypothesis traps limit relevant results

Different concurrent explanations may explain the limited number of studies that produce significant statistical differences. One plausible explanation, known as the 'omitted variable bias', notes that outcome measures such as hospital readmissions and length of stay are moderated by other confounding variables that are usually not captured in large-sample studies. Conversely, the same outcomes cannot be easily observed in smaller-sample in-depth studies, due to the impact of sample size in hypothesis testing.

Another explanation is the 'outcome selection trap', which refers to the practice of identifying relevant and easy-to-collect outcomes (therefore generating less measurement error) in intervention studies. However, those outcomes could be considered excessively distant from the nature of the interventions. For instance, valuable low-impact interventions such as follow-up phone calls should not be assessed in terms of reduction of hospitalisation, but in terms of other subjective measures such as quality of life or process-related outputs such as correct use of medication.

A third explanation is the 'causality gap', which refers to the inability of many of those interventions to establish a clear theoretical causal connection between, on one hand, activities and resources and, on the other, outputs and outcomes. This includes an inability to estimate the proportion of resources, time and activities needed to achieve specific measurable changes, such improvements in health status.

A fourth explanation is the 'timeframe gap', whereby studies do not analyse effects over a sufficient long time period, where relevant changes might be more easily observable over lengthier time frames.

For peer review only - http://bmjopen.bmj.com/site/about/guidelines.xhtml

The diversity and limitations of interventions

The review included different interventions focusing on reducing the ADH of older people, varying from a range of complex designs such as hospital-at-home programmes to simpler ones such as medication reviews. These interventions occurred over different lengths of time, including short-term transitional care services and longer ones such as hospital-at-home assistance and case-management interventions. The number of caregivers, the permanent, periodical or sporadic nature of their work, and the diversity in terms of specialisations varies significantly.

All fifteen studied interventions were based almost entirely within the health system and were provided by a wide range of health specialists. With the exception of three studies [12–14], there was no integration between mainstream health services and other agencies, such as housing services or social care.

At the same time, there was an absence of preventive interventions. All the quasi-experiments and RCT review interventions viewed the hospital setting as the 'initial status' of the intervention. Only one study emphasised preventive care through regular clinical monitoring [21]. This is especially relevant for interventions that focus on a wide range of older people with different health statuses and different degrees of functional dependency.

Assessing interventions with the 'avoidable displacement from home' framework

The ADH framework provides insights in terms of the assessment of integrated care interventions. As mentioned above, this includes three key elements: person-centredness, place-based care and sustainability. The only interventions presenting statistically significant results were case management services [20,21], characterised by a person-centred approach that focusses on individual rehabilitation and care plans.

Additionally, interventions that focus on place-based care can be evaluated in terms of the extent to which the service provision was fragmented, and the availability of services at home -this applies to all the studies presented in this paper. It was not possible to determine the degree of service fragmentation of each intervention, although some of them (e.g. medication review) fell short in terms of integration of care.

Finally, in terms of assessing intervention sustainability, many were short-term, which was not necessarily compatible with patient needs. Finally, only three studies provide any element of economic analysis [14,16,25].

For peer review only - http://bmjopen.bmj.com/site/about/guidelines.xhtml

For peer review only

Comparison with previous literature reviews

Three previous systematic reviews [26–28] also examine the effectiveness of interventions to prevent hospitalisations based on home care and also demonstrate the limitations of current impact evidence. Additionally, Huntley et al [29] review evidence about alternatives to acute care, such as paramedic/emergency department-based interventions and community hospitals. Their main finding is that impact evidence is limited by a lack of systematic data on outcomes and costs for patients, health professionals and carers.

Limitations of this review

This review looks at ADH interventions in home settings. It does not consider other settings, including those that may be applied in hospitals or in nursing homes, to reduce ADH. Interventions in these other settings should be the focus of future reviews.

All the articles but one in this review studied health care systems in high-income countries, even though low or middle-income countries contain a larger number of older people with health and care needs. This may reflect the limited number of languages included in the study, although this is unlikely to have excluded a large number of studies.

Empirical evidence in this area is predominately from quasi-experiments and observational studies, while studies with low risk of bias were an exception.

CONCLUSIONS AND IMPLICATIONS

The review was not able to identify robust impact evidence either in terms of quantity or quality from the studies presented. Therefore, the strength of the evidence cannot be considered sufficiently strong to inform policy or interventions seeking to reduce ADH. The paucity of evidence does not result from the limited number of potentially relevant interventions. Rather, it reflects the complexity of these interventions and a lack of systematic data collection. As such, this review identifies an urgent need for systematic monitoring and data management, as well as further enhancement of methods for evaluating complex interventions.

Contributors: LS: lead systematic reviewer conducting all stages of the review, and was responsible for the initial draft of protocol and draft of paper. JB: second reviewer; contributing to discussion as the review progressed; commenting and editing on the drafts of the paper. PLS: secured funding, idea of current work, third reviewer and contributing to discussion as the review progressed; commenting and editing on the drafts of the paper.

For peer review only - http://bmjopen.bmj.com/site/about/guidelines.xhtml

Funding: This paper presents research funded by Eastern ARC Consortium and Medical Research Council (grant reference number MR/R024219/1).

Disclaimer: The views expressed are those of the authors and not necessarily those of the Eastern ARC Consortium and Medical Research Council

Competing interests: None declared.

Patient consent for publication: Not required

Provenance and peer review: Not commissioned; externally peer reviewed.

Data sharing statement: No additional data are available.

Open access: This is an open access article distributed in accordance with the Creative Commons Attribution 4.0 Unported (CC BY 4.0) license, which permits others to copy, redistribute, remix, transform and build upon this work for any purpose, provided the original work is properly cited, a link to the licence is given, and indication of whether changes were made. See: https://creativecommons.org/ licenses/by/4.0/.

REFERENCES

1 United Nations. Department of Economic and Social Affairs. Population Division. World Population Ageig 2017 - Highlights (ST/ESA/SER.A/397). 2017. doi:10.1049/el:20000788

2 Souza DK de, Peixoto SV. Estudo descritivo da evolução dos gastos com internações hospitalares por condições sensíveis à atenção primária no Brasil, 2000-2013*. *Epidemiol e Serviços Saúde* 2017;**26**:285–94. doi:10.5123/s1679-49742017000200006

3 Wright PN, Tan G, Iliffe S, *et al.* The impact of a new emergency admission avoidance system for older people on length of stay and same-day discharges. *Age Ageing* 2013;**43**:116–21. doi:10.1093/ageing/aft086

4 Charles A, Wenzel L, Kershaw M, *et al.* A year of integrated care systems: reviewing the journey so far. 2018. https://www.kingsfund.org.uk/sites/default/files/2018-09/Year-of-integrated-care-systems-reviewing-journey-so-far-full-report.pdf

5 Briggs A, Araujo de Carvalho I. Actions required to implement integrated care for older people in the community using the World Health Organization ' s ICOPE

For peer review only - http://bmjopen.bmj.com/site/about/guidelines.xhtml

approach : A global Delphi consensus study. *PLoS One* 2018;**13**:1–16.

6 McCormack B. Person-centredness in gerontological nursing: an overview of the literature. *J Clin Nurs* 2004;**13**:31–8. doi:10.1016/j.maturitas.2016.07.010

7 Ham CAH. Place-based Health Systems. *King's Fund* 2015;:1–50.http://www.kingsfund.org.uk/sites/files/kf/field/field_publication_file/Place-based-systems-of-care-Kings-Fund-Nov-2015_0.pdf

8 de Bruin SR, Stoop A, Billings J, *et al.* The SUSTAIN Project: A European Study on Improving Integrated Care for Older People Living at Home. *Int J Integr Care* 2018;**18**:1–12. doi:10.5334/ijic.3090

9 Higgins JP, Green S. *Cochrane Handbook for Systematic Reviews of Interventions*. 2011. doi:10.1002/9780470712184.ch5

10 Meret-Hanke LA. Effects of the program of all-inclusive care for the elderly on hospital use. *Gerontologist* 2011;**51**:774–85. doi:10.1093/geront/gnr040

11 Courtney M, Edwards H, Chang A, *et al.* Fewer emergency readmissions and better quality of life for older adults at risk of hospital readmission: A randomized controlled trial to determine the effectiveness of a 24-week exercise and telephone follow-up program. *J Am Geriatr Soc* 2009;**57**:395–402. doi:10.1111/j.1532-5415.2009.02138.x

12 Hendrix C, Tepfer S, Forest S, *et al.* Transitional Care Partners: a hospital-to-home support for older adults and their caregivers. *J Am Assoc Nurse Pract* 2013;**25**:407–14. doi:10.1111/j.1745-7599.2012.00803.x

13 Stranges PM, Marshall VD, Walker PC, *et al.* A Multidisciplinary Intervention for Reducing Readmissions Among Older Adults in a Patient-Centered Medical Home. *Clinical* 2015;**21**:106–13.

14 Sahota O, Pulikottil-Jacob R, Marshall F, *et al.* The Community In-reach Rehabilitation and Care Transition (CIRACT) clinical and cost-effectiveness randomisation controlled trial in older people admitted to hospital as an acute medical emergency. *Age Ageing* 2017;**46**:26–32. doi:10.1093/ageing/afw149

15 Young JB, Robinson M, Chell S, *et al.* A whole system study of intermediate care services for older people. *Age Ageing* 2005;**34**:577–83. doi:10.1093/ageing/afi179

For peer review only - http://bmjopen.bmj.com/site/about/guidelines.xhtml

16 Harris R, Ashton T, Broad J, *et al.* The effectiveness, acceptability and costs of a hospital-at-home service compared with acute hospital care: a randomized controlled trial. *J Heal Serv Res Policy* 2005;**10**:158–66.http://onlinelibrary.wiley.com/o/cochrane/cleed/articles/NHSEED-22005007671/frame.html

17 Robinson TE, Zhou L, Kerse N, *et al.* Evaluation of a New Zealand program to improve transition of care for older high risk adults. *Australas J Ageing* 2015;**34**:269–74. doi:10.1111/ajag.12232

18 Ong KY, Cheen MHH, Chng JSG, *et al.* Effectiveness of a multidisciplinary home-based medication review program in reducing healthcare utilization among older adult Singaporeans. *Geriatr Gerontol Int* 2017;**17**:302–7. doi:10.1111/ggi.12706

19 Low LL, Vasanwala FF, Ng LB, *et al.* Effectiveness of a transitional home care program in reducing acute hospital utilization: A quasi-experimental study. *BMC Health Serv Res* 2015;**15**:1–8. doi:10.1186/s12913-015-0750-2

20 de Stampa M, Vedel I, Buyck J-F, *et al.* Impact on hospital admissions of an integrated primary care model for very frail elderly patients. *Arch Gerontol Geriatr* 2014;**58**:350–5. doi:10.1016/J.ARCHGER.2014.01.005

21 Caplan GA, Williams AJ, Daly B, *et al.* A Randomized , Controlled Trial of Comprehensive Geriatric Elderly from the Emergency Department - The DEED II Study. *J Am Geriatr Soc* 2004;**52**:1417–23.

22 Rytter L, Jakobsen HN, Rønholt F, *et al.* Comprehensive discharge follow-up in patients' homes by GPs and district nurses of elderly patients. *Scand J Prim Health Care* 2010;**28**:146–53. doi:10.3109/02813431003764466

23 Espinel-Bermúdez MC, Sánchez-García S, Juárez-Cedillo T, *et al.* Impacto de un programa de atención domiciliaria al enfermo crónico en ancianos: Calidad de vida y reingresos hospitalarios. *Salud Publica Mex* 2011;**53**:17–25. doi:10.1590/S0036-36342011000100004

24 Leung DYP, Lee DTF, Lee IFK, *et al.* The effect of a virtual ward program on emergency services utilization and quality of life in frail elderly patients after discharge: A pilot study. *Clin Interv Aging* 2015;**10**:413–20. doi:10.2147/CIA.S68937

For peer review only

25 Graves N, Courtney M, Edwards H, *et al.* Cost-Effectiveness of an Intervention to Reduce Emergency Re-Admissions to Hospital among Older Patients. *PLoS One* 2009;**4**:e7455. doi:10.1371/journal.pone.0007455

26 Shepperd S, Iliffe S, Doll H, *et al.* Admission avoidance hospital at home. *Cochrane Database Syst Rev* 2016;**2016**. doi:10.1002/14651858.CD007491.pub2

27 Oeseburg B, Wynia K, Middel B, *et al.* Effects of Case Management for Frail Older People or Those With Chronic Illness. A Systematic Review. *Nurs Res* 2009;**58**:201–10.

28 Linertová R, García-Pérez L, Vázquez-Díaz JR, *et al.* Interventions to reduce hospital readmissions in the elderly: In-hospital or home care. A systematic review. *J Eval Clin Pract* 2011;**17**:1167–75. doi:10.1111/j.1365-2753.2010.01493.x

29 Huntley AL, Chalder M, Shaw ARG, *et al.* A systematic review to identify and assess the effectiveness of alternatives for people over the age of 65 who are at risk of potentially avoidable hospital admission. *BMJ Open* 2017;**7**:1–7. doi:10.1136/bmjopen-2017-016236

For peer review only

For peer review only - http://bmjopen.bmj.com/site/about/guidelines.xhtml

Legends:

Figure 1. PRISMA flow diagram of literature review

For peer review only

For peer review only - http://bmjopen.bmj.com/site/about/guidelines.xhtml

APPENDIX 1: SEARCH STRATEGY

“home” OR “home hospital” OR “out-patient facility” OR “hospital in home” OR “Home care” OR “Domiciliary care” OR “outreach programme” OR “hospital outreach” OR “comprehensive elderly care” OR “home hospitalisation" AND “Frail elderly” OR “Elderly, Frail” OR “Functionally-impaired Elderly” OR “Aged OR elderly” OR “Aged, 80 and over” OR “Centenarians” OR “Nonagenarians” OR “Octogenarians" AND “Involuntary hospitalisation” OR “Avoidable hospitalisation" OR “Patient admission” OR “Voluntary Admission” OR “Patient Readmission” OR “30 Day Readmission” OR “Thirty Day Readmission” OR “Hospital Readmissions” OR “Readmissions, Hospital".

APPENDIX 2: DATA EXTRACTION FORM

Title
Author
Year
Methods
Participants
Patients
Classification of intervention
Intervention
Team
Person centeredness
Sustainability
Preventive aspect
Outcomes
Results
Conclusion

For peer review only

APPENDIX 3: RISK BIAS

Author	Random sequence generation (selection bias)	Allocation concealment (selection bias)	Blinding of participant and personnel (performance bias)	Blinding of outcome assessment (detection bias)	Incomplete outcome data (attrition bias)	Selective reporting bias
Caplan et al	Unclear risk (block randomisation)	Low risk (opaque envelopes)	Higk risk ("almost half conducted by a research assistant, with the rest performed by different members of the multidisciplinary team")	Low risk. Baseline characteristics are similar	"Unclear (10.0% of intervention participants and 13.0% of control participants withdrew from the study. The authors did not report reasons for withdrawal.)"	"High risk (Only data at 1 and 18 months were reported, yet measurements were taken at 1, 3, 6, 12 and 18 months)"
Courtney et al	Low risk (computer-generated)	High risk (not mentioned)	Unclear risk (not mentioned)	Low risk. Baseline characteristics are similar	High risk (attrition and 28% lack of compliance)	Low risk. Main outcomes are reported
de Stampa el al	High risk. 'Patients were recruited consecutively until the desired sample sizes were achieved.'		Low risk. Interventions occurs in different geographical areas	Low risk. Similar baseline characteristics	Low risk. Attrition is similar in both groups (deceased or institutionalised)	Low risk. Results are reported as stated in protocol
Espinel-Bermudez et al	High risk. Unclear criteria of selection (mentions quota and convenience sampling)		Low risk. (Two different hospitals, doing ordinary activities)	High risk. Baseline 'Length of stay' and comorbidity index differs between groups.	High risk. Unbalanced groups leads to different attrition loss.	Unclear
Harris et al	Low risk (computer-generated)	Unclear. Do not mention allocation process	Low risk ("Interviews by trained researchers who were not involved in patient care, but who were	Low risk (baseline results similar)	Unclear. Loss to follow-up: hospital at home: 14/143; hospital: 17/142	High risk (no mention to registered protocol)

			not blinded as to the intervention group")			
Hendrix et al	DNA	DNA	DNA	DNA	Low risk	High risk (absence of statistical analysis)
Leung et al	DNA	DNA	DNA	DNA	Low risk (no loss of patients)	High risk (statistical analysis incomplete)
Low et al	DNA	DNA	DNA	DNA	Low risk. Loss of 3 out of 262 patients	High risk. Does not control for covariables
Meret-Hanke	Low risk. "First, propensity score matching assumes that outcomes are independent of treatment assignment given an observable set of covariates (Dehejia & Wahba, 2002; Rosenbaum & Rubin, 1983). Second, the propensity score distributions of the treatment and comparison groups are assumed to overlap (Rosenbaum & Rubin, 1983). To satisfy these assumptions, we included covariates that strongly affect both the treatment decision and the outcome to effectively reduce bias (Smith & Todd, 2005)."		Low risk. Retrospective analysis	High risk. "For instance, individuals who enroll in PACE could be more averse to using hospitals than other individuals." "In addition, although we were able to match on most individual characteristics, we were unable to match on market, geographic, and historical factors."	Low risk. Different attrition bias analysis performed (baseline characteristics lost to attrition, effect of cases on outcomes)	Unclear

**Ong et al**	DNA	DNA	DNA	DNA	Low risk. 12% of deaths	Low risk. Main outcomes are reported
**Robinson et al**	Low risk. Threshold for RD is the minimum required for treatment ('risks core where the patients begin to receive the intervention(20% risk)')		Low risk. Quasi-experimental design (RD and ITS)	Low risk. Quasi-experimental design (RD and ITS)	Low risk. 'From this target group we identified eligible 'High Risk' patients using a predictive risk model (PRM) that could be run early in the patient's admission'	Low risk ("We measured whether the intervention was delivered according to the initial plan. In particular we measured whether patients received the discharge pharmacy and the nurse telephone follow-up services using program administrative records")
**Rytter et al**	Low risk (computer generated)	Low risk (opaque envelopes)	High risk (not blinded to GP and nurses)	Low risk (baseline characteristics similar)	Unclear risk (Dropouts of lower functional ability patients)	Low risk. Main outcomes reported.

**Sahota et al**	Low risk (computer-generated)	Low risk (The randomisations will be requested through a PC with internet explorer and internet access. The system is located on a dedicated secure server within the University of Nottingham)	Low risk. Protocol: The RA collecting data and the research team analysing the data will be blinded to treatment allocation. The participant and ward staff will not be blinded to treatment allocation as the treating therapists will be liaising closely with ward staff to ensure optimal patient care. The three-month follow-up data will be collected by an RA blinded to treatment allocation.	Low risk (The groups appeared well balanced at baseline)	Low risk (212 participant, 106 in each arm)	Low risk (similar outcomes as protocol)
**Stranges et al**	DNA	DNA	DNA	Low risk (Propensity Score Matching)	Low risk (retrospective analysis)	Low risk. Main outcomes are reported
**Young et al**	High risk. Patients were recruited consecutively		Unclear. Evaluation occurs within policy change	Low risk. Baseline characteristics are similar	Unclear. Mortality rate is similar after 1 year	Low risk. Main outcomes are reported

DNA: Do not apply

APPENDIX 4: PRISMA CHECKLIST

Section/topic	#	Checklist item	Reported on page #
TITLE			
Title	1	Identify the report as a systematic review, meta-analysis, or both.	1
ABSTRACT			
Structured summary	2	Provide a structured summary including, as applicable: background; objectives; data sources; study eligibility criteria, participants, and interventions; study appraisal and synthesis methods; results; limitations; conclusions and implications of key findings; systematic review registration number.	1
INTRODUCTION			
Rationale	3	Describe the rationale for the review in the context of what is already known.	1-2
Objectives	4	Provide an explicit statement of questions being addressed with reference to participants, interventions, comparisons, outcomes, and study design (PICOS).	2
METHODS			
Protocol and registration	5	Indicate if a review protocol exists, if and where it can be accessed (e.g., Web address), and, if available, provide registration information including registration number.	2
Eligibility criteria	6	Specify study characteristics (e.g., PICOS, length of follow-up) and report characteristics (e.g., years considered, language, publication status) used as criteria for eligibility, giving rationale.	2
Information sources	7	Describe all information sources (e.g., databases with dates of coverage, contact with study authors to identify additional studies) in the search and date last searched.	2
Search	8	Present full electronic search strategy for at least one database, including any limits used, such that it could be repeated.	2-3 AND APPENDIX
Study selection	9	State the process for selecting studies (i.e., screening, eligibility, included in systematic review, and, if applicable, included in the meta-analysis).	3
Data collection process	10	Describe method of data extraction from reports (e.g., piloted forms, independently, in duplicate) and any processes for obtaining and confirming data from investigators.	3
Data items	11	List and define all variables for which data were sought (e.g., PICOS, funding sources) and any assumptions and simplifications made.	APPENDIX
Risk of bias in individual studies	12	Describe methods used for assessing risk of bias of individual studies (including specification of whether this was done at the study or outcome level), and how this information is to be used in any data synthesis.	3
Summary measures	13	State the principal summary measures (e.g., risk ratio, difference in means).	DNA
Synthesis of results	14	Describe the methods of handling data and combining results of studies, if done, including measures of consistency (e.g., I^2) for each meta-analysis.	DNA

For peer review only

Risk of bias across studies	15	Specify any assessment of risk of bias that may affect the cumulative evidence (e.g., publication bias, selective reporting within studies).	APPENDIX
Additional analyses	16	Describe methods of additional analyses (e.g., sensitivity or subgroup analyses, meta-regression), if done, indicating which were pre-specified.	DNA
RESULTS			
Study selection	17	Give numbers of studies screened, assessed for eligibility, and included in the review, with reasons for exclusions at each stage, ideally with a flow diagram.	4
Study characteristics	18	For each study, present characteristics for which data were extracted (e.g., study size, PICOS, follow-up period) and provide the citations.	5-8
Risk of bias within studies	19	Present data on risk of bias of each study and, if available, any outcome level assessment (see item 12).	APPENDIX
Results of individual studies	20	For all outcomes considered (benefits or harms), present, for each study: (a) simple summary data for each intervention group (b) effect estimates and confidence intervals, ideally with a forest plot.	DNA
Synthesis of results	21	Present results of each meta-analysis done, including confidence intervals and measures of consistency.	DNA
Risk of bias across studies	22	Present results of any assessment of risk of bias across studies (see Item 15).	9 AND APPENDIX
Additional analysis	23	Give results of additional analyses, if done (e.g., sensitivity or subgroup analyses, meta-regression [see Item 16]).	DNA
DISCUSSION			
Summary of evidence	24	Summarize the main findings including the strength of evidence for each main outcome; consider their relevance to key groups (e.g., healthcare providers, users, and policy makers).	9-13
Limitations	25	Discuss limitations at study and outcome level (e.g., risk of bias), and at review-level (e.g., incomplete retrieval of identified research, reporting bias).	14
Conclusions	26	Provide a general interpretation of the results in the context of other evidence, and implications for future research.	13-14
FUNDING			
Funding	27	Describe sources of funding for the systematic review and other support (e.g., supply of data); role of funders for the systematic review.	14

From: Moher D, Liberati A, Tetzlaff J, Altman DG, The PRISMA Group (2009). Preferred Reporting Items for Systematic Reviews and Meta-Analyses: The PRISMA Statement. PLoS Med 6(7): e1000097. doi:10.1371/journal.pmed1000097

For peer review only

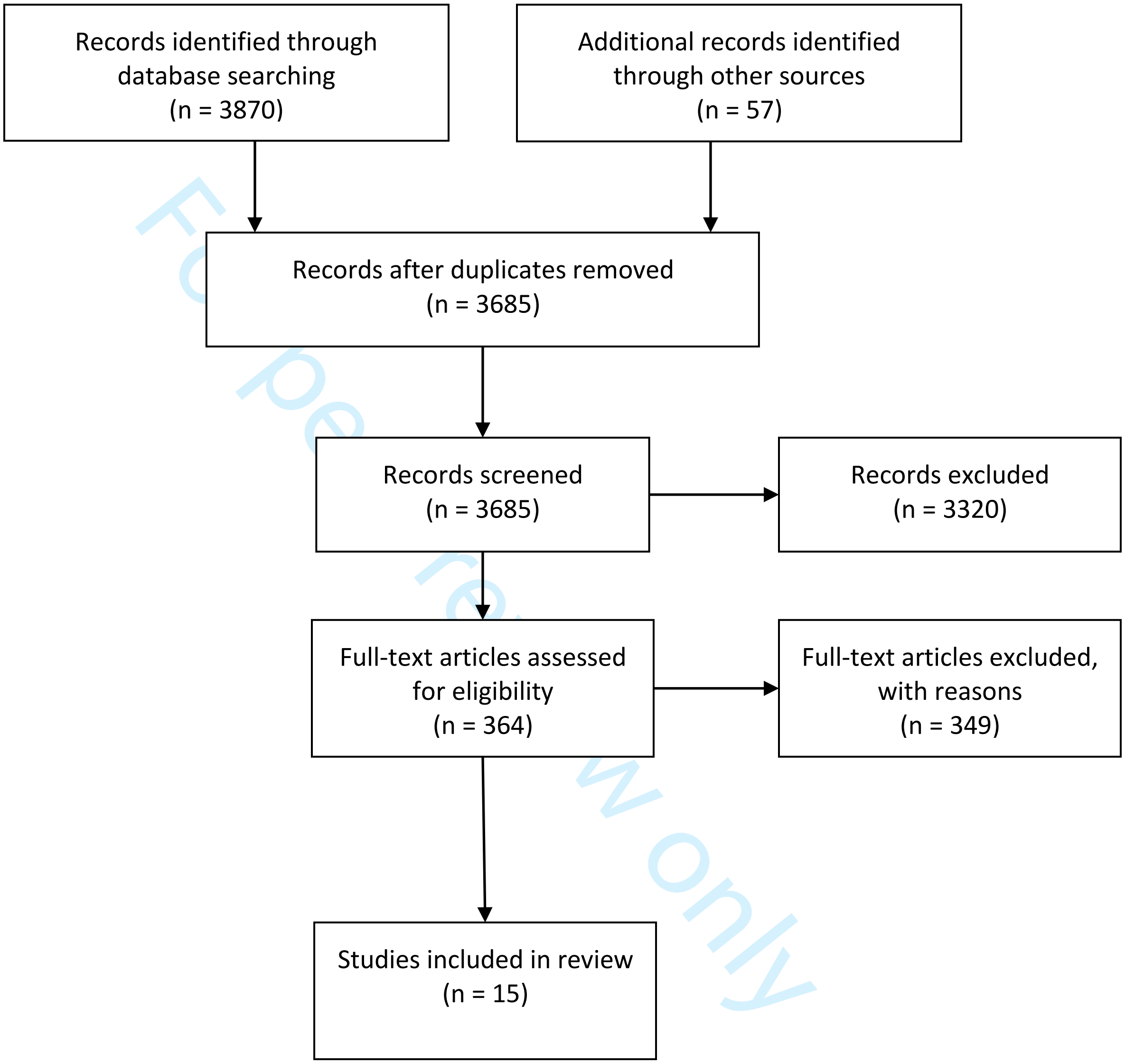

From: Moher D, Liberati A, Tetzlaff J, Altman DG, The PRISMA Group (2009). *P*referred *R*eporting *I*tems for *S*ystematic Reviews and *M*eta-*A*nalyses: The PRISMA Statement. PLoS Med 6(7): e1000097. doi:10.1371/journal.pmed1000097

For more information, visit www.prisma-statement.org.

BMJ Open

Multidisciplinary interventions for reducing the avoidable displacement from home of frail older people: a systematic review

Journal:	*BMJ Open*
Manuscript ID	bmjopen-2019-030687.R1
Article Type:	Original research
Date Submitted by the Author:	04-Jul-2019
Complete List of Authors:	Sempé, Lucas; University of East Anglia, School of International Development<br>Billings, Jenny; University of Kent, Centre for Integrated Care Research<br>Lloyd-Sherlock, Peter; University of East Anglia, School of International Development
**Primary Subject Heading**:	Health policy
Secondary Subject Heading:	Patient-centred medicine, Health policy
Keywords:	healthcare, older people, social care, systematic review, avoidable displacement from home

SCHOLARONE™
Manuscripts

Multidisciplinary interventions for reducing the avoidable displacement from home of frail older people: a systematic review

Lucas Sempé,[1] Jenny Billings,[2] Peter Lloyd-Sherlock[1]

[1] School of International Development, University of East Anglia, Norwich, NR4 7TJ, UK

[2] Centre for Health Services Studies, University of Kent, Canterbury, CT2 7NZ, UK

Corresponding author: Lucas Sempé. E-mail l.sempe@uea.ac.uk / Telephone: +447907994151

Word count: 4735

For peer review only

For peer review only - http://bmjopen.bmj.com/site/about/guidelines.xhtml

ABSTRACT

Objectives: To synthesise existing literature on interventions addressing a new concept of avoidable displacement from home for older people with multimorbidity or frailty. The review focused on home-based interventions by any type of multidisciplinary team aimed at reducing avoidable displacement from home to hospital settings. A second objective was to characterise these interventions to inform policy.

Design: A systematic search of the main bibliographic databases was conducted to identify studies relating to interventions addressing avoidable displacement from home for older people. Studies focusing on one specific condition or interventions without multidisciplinary teams were excluded. A narrative synthesis of data was conducted, and themes were identified by using an adapted thematic framework analysis approach.

Results: The search strategy was performed using the following electronic databases: the American National Library of Medicine and the National Institutes of Health (PubMed), Scopus, Cochrane Library (Central and CDRS), CINAHL, Social Care Online, Web of Science as well as the database of the Latin American and Caribbean Health Sciences Literature (LILACS). The database search was done in September 2018 and completed in October 2018. Overall 3927 articles were identified and 364 were retained for full text screening. Fifteen studies were included in the narrative review. Four themes were identified and discussed: (1) types of interventions; (2) composition of teams; (3) intervention effectiveness; and (4) types of outcomes. Within intervention types, three categories of care types were identified; transitional care, case-management services and hospital at home. Each individual article was assessed in terms of risk of bias following Cochrane Collaboration guidelines.

Conclusions: The review identified some potential interventions and relevant topics to be addressed in order to develop effective and sustainable interventions to reduce the avoidable displacement from home of older people. However the review was not able to identify robust impact evidence, either in terms of quantity or quality from the studies presented. As such, the available evidence is not sufficiently robust to inform policy or interventions for reducing avoidable displacement from home. This finding reflects the complexity of these interventions and a lack of systematic data collection.

Keywords: avoidable displacement from home, healthcare, older people, social care, systematic review

STRENGTHS AND LIMITATIONS OF THIS STUDY

- This is a timely systematic review of interventions involving integrated care for older people, situated within a policy framework that focuses on a new concept of avoidable displacement from home.
- This review examines interventions addressing a significant public health and social care challenge.
- Evidence in this area is predominately from quasi-experiments and observational studies with a number of studies presenting unclear results and ambiguity regarding the effectiveness of the interventions on hospital and emergency department admissions and readmissions.

For peer review only - http://bmjopen.bmj.com/site/about/guidelines.xhtml

INTRODUCTION

Rationale

Between 2017 and 2050 the global population of people aged 60 years or over is expected to more than double, reaching 2.1 billion people. In 2015 around two thirds of this population lived in low or middle-income countries, rising to 80% by 2050 [1]. Later life is associated with an increased risk of frailty, poor health and functional limitation[2]. In this paper, frailty refers to a distinctive health state related to the ageing process in which multiple body systems gradually lose their in-built reserves [3]. Population ageing creates challenges for both social services and health systems, including rising numbers of hospitalisations, many of which are potentially avoidable [4,5]. Responses to these challenges include an increased emphasis on interventions to integrate different forms of health and social care provision, working across different care settings [6]. There are several definitions of integrated care: this paper follows Kodner and Kyriacou's [7] who define it as a "… set of techniques and organisational models designed to create connectivity, alignment and co-ordination within and between the cure and care sectors at the funding, administrative and/or provider levels…. for patients with complex problems". While different degrees of integration are possible, all by definition require the cooperation of multidisciplinary professional teams working together to deliver appropriate care. Multidisciplinary interventions to promote and support integrated care are now widely advocated at many policy levels. It is claimed, however, that the effectiveness of these approaches is inconsistent across care settings and different interventions [8].

A new framework: avoidable displacement from home

The concept of avoidable displacement from home and an associated policy framework are currently being developed and validated by the authors as part of an MRC Newton funded project 'Improving the effectiveness and efficiency of Health and social care services for vulnerable Older Brazilians' (IHOB) (2018-2021) [9]**.** This review represents one aspect of the validation process.

Given the wide diversity of integrated care interventions and their complex interactions with other parts of health and social care systems, it is important to establish clear parameters about where they are situated in relation to different care settings and to clarify outcomes of interest. To this end, 'avoidable displacement from home' may provide a helpful framework, by encompassing a number of effects: hospital (re)admissions that are amenable to prevention or which are suited to outpatient care, excessive hospital stays and unnecessary admissions into residential care homes. More broadly, an avoidable displacement from home can be understood as the consequence of failures to deliver proper care that permits older people to remain in their homes for as long as possible when this is in their best interest. It represents a comprehensive approach to address health and social care as part of a single, integrated system.

Interventions to reduce avoidable displacement from home should aim to incorporate three core values: person-centredness [10], place-based care [11] and sustainability [12]. In this way, the concept may encourage policy-makers to go beyond an exclusive focus on health system efficiency (such as reducing unnecessary hospitalisations) towards a wider approach that seeks to reconcile health and social service provision with the needs and wishes of both older people and their carers.

For peer review only - http://bmjopen.bmj.com/site/about/guidelines.xhtml

Objective

Our specific objective was to conduct a systematic review to identify interventions addressing displacement from home of older people with multimorbidity or frailty. The review focused on studies with the following characteristics: home-based interventions by any type of multidisciplinary team that aimed at reducing avoidable displacement from home in older people with multimorbidity or frailty. A second objective was to characterise those interventions in order to inform policy.

METHODS

Protocol and registration

The protocol for the systematic review was listed on the PROSPERO prospective register on 3 September 2018. The registration number is CRD42018108116 (supplementary material).

Information sources and searches

This is a systematic review of articles indexed in the following electronic databases: the American National Library of Medicine and the National Institutes of Health (PubMed), Scopus, Cochrane Library (Central and CDRS), CINAHL, Social Care Online, Web of Science as well as the database of the Latin American and Caribbean Health Sciences Literature (LILACS). We also examined the reference lists of the selected studies.

The search was conducted without any language restriction and focused on articles published after the year 2000. The review only included articles published in Portuguese, English, and Spanish, because of our familiarity with these languages. The protocol for the related search terms was completed in July 2018 while the full database search was performed in September 2018 and the articles were moved into Mendeley 1.18. The duplicates have been deleted.

Search strategy

An initial scoping of the literature was conducted at inception of the study and those findings were used to define the search strategy. In order to take an inclusive approach we included a variety of key search terms, utilising the scope of Medical Subject Headings (MeSH) for each of them. The terms linked three different topics, namely: 'home-based care' interventions, and terms to identify 'older adults' in conjunction with terms used as proxies of preventive outcomes ("Involuntary hospitalisation", "Avoidable hospitalisation", "Patient admission" or "Voluntary Admission") and readmissions ("Patient Readmission", "30 Day Readmission", "Thirty Day Readmission", "Hospital Readmissions" or "Readmissions, Hospital"). The term 'multidisciplinary teams' or related terms were not used in the search to prevent restricting the results. The search syntax and data extraction form are provided in Appendix 1 and 2.

Eligibility criteria

Following the review objectives, any type of quantitative study (RCTs, quasi-experiments and observational data) that fitted our criteria was included in the review process. The eligibility criteria guiding the search enabled the selection of publications specifically focusing on older people (defined as aged over 60 years) who received any kind of home-based intervention by a multidisciplinary team. A multidisciplinary team was defined as a formal team of two or more people from different professions

For peer review only - http://bmjopen.bmj.com/site/about/guidelines.xhtml

working together to deliver their service. In order to assess any effect on avoidable displacement from home, the studies needed to record at least one of the following outcomes: hospital length of stay, hospital or emergency department admissions or readmissions.

Study selection and data extraction process

The data extraction process had two stages. Two authors (LS, JB) independently screened all titles and abstracts identified from searches to determine which met the inclusion criteria. They also independently screened full-text articles for inclusion or exclusion, with discrepancies resolved by discussion and by consulting a third author (PLS). All potentially relevant papers excluded from the review at this stage are listed as excluded studies, with reasons provided in the Characteristics of Excluded Studies table. A data extraction form was developed to extract and appraise each study according to the aims of the study.

Assessment of risk of bias of individual studies

Risk of bias was assessed using the Cochrane Collaboration tool [13] and was rated as low, unclear, or high. Ratings of low risk of bias indicate high confidence in study findings. Ratings of unclear risk of bias indicate the presence of probable bias or flaws that raise reservations about study findings. High risk of bias are indicators of poor study quality, and results are considered with caution. One author (LS) assessed the risk of bias of the included studies, which was reviewed by both additional authors (JB, PLS). Any disagreements were resolved by discussion to reach consensus.

Synthesis of results

Thematic analysis was conducted within and across categories to identify key themes. All authors deliberated and agreed on these themes, as well as their validity and implications. Since one objective of this review is to inform policy, descriptive information about all interventions is presented and discussed in the results section. However, only those presenting low or unclear risk of bias are summarised and discussed in terms of their effectiveness. Due to the heterogeneity of reported outcomes and risk of bias assessed in individual studies, a meta-analysis was not feasible.

Four themes were identified: (1) types of interventions; (2) composition of teams; (3) intervention effectiveness; and (4) types of outcomes. Within intervention types, three categories of care types were identified; transitional care, case-management services; and hospital at home. It is important to notice that these categories were not mutually exclusive and that some degree of overlap of the interventions' characteristics existed among the categories. These connections are highlighted in the results section.

Patient and public involvement

Patients and the public were not involved in the design or analysis of this review.

Systematic review reporting items

This Systematic Review follows PRISMA's checklist of preferred reporting Items for Systematic Reviews, detailed in Appendix 3.

For peer review only - http://bmjopen.bmj.com/site/about/guidelines.xhtml

RESULTS

Study selection

A total of 3927 articles were identified. After screening titles and abstracts, 364 full-texts were reviewed, of which fifteen articles met the inclusion criteria (Figure 1).

For peer review only

For peer review only - http://bmjopen.bmj.com/site/about/guidelines.xhtml

(Insert Figure 1)

Study characteristics

Regarding types of interventions, the systematic review identified 15 interventions, which were catalogued into three different care types: transitional care (n=8), case-management services (n=4), and interventions focusing on hospital-at-home (n=3).

Five studies were RCTs, eight were quasi-experiments (four prospective controlled pre-post design and one retrospective controlled pre-post design) and three were retrospective non-controlled observational studies. Four interventions took place in the United States [14–17], while two interventions were set in the United Kingdom [18,19], New Zealand [20,21], and Singapore [22,23]. Additionally, there was one intervention each in France [24], Australia [25], Denmark [26], Mexico [27] and Hong Kong [28]. See Table 1 for a summary of study characteristics.

For peer review only

For peer review only - http://bmjopen.bmj.com/site/about/guidelines.xhtml

Table 1. Summary of studies

Study	*Year*	*Methods*	*Number of participants*	*Demographics*	*Classification of intervention*	*Intervention*	*Team*	*Outcomes*	*Quality and bias assessment*
Caplan et al	2004	RCT	739	>75	Case-management	Comprehensive geriatric assessment and multidisciplinary intervention on older patients sent home from the emergency department (ED). A nurse formulates a care plan, initiates urgent interventions and referrals, and presents the patient's history at a weekly interdisciplinary team meeting at which further interventions or referrals can be ordered.	Meetings attended by a geriatrician or a geriatric registrar, nurses, physiotherapists, and occupational therapists	All hospital admissions within 30 days<br>Elective and emergency admissions (days to first emergency admission)<br>Nursing home admissions<br><br>Mortality<br><br>Physical function (Barthel Index)<br>Instrumental activities of daily living<br>Cognitive function	Unclear risk of bias
Courtney et al	2009	RCT and economic study (in additional paper)	128	>65	Transition care	Comprehensive nursing and physiotherapy assessment and individualised programme of exercise strategies. Home visit by a nurse. Telephone follow-up commencing in the hospital and continuing for 24 weeks after discharge.	Nurse and physiotherapist	Hospital readmissions and emergency GP visits	Unclear quality and risk of bias
de Stampa el al	2014	Prospective controlled pre-post design	428	>64	Case-management	COPA. Multi-disciplinary comprehensive geriatric needs assessment. This includes an individual care plan, care management programmes, evidence-based protocols, and regular reassessments of patient needs	Case managers (geriatric nurses), primary care physician and a psychologist	Unplanned hospitalisation (via ED)<br>Any hospitalisation (1 year)<br><br>Instrumental Activity of Daily Living<br>Activity of Daily Living Hierarchical<br>Cognitive Performance<br>Depression Rating<br>Aggressive Behaviour<br>Pain Scale	Unclear risk of bias
Espinel-Bermudez et al	2011	prospective controlled pre-post design	70	>60	Hospital at home	Programme of domiciliary attention for chronically ill patients. Two educational sessions with patient carers. Home visits, provision of medical equipment and medication.	Two teams. The first in based in the hospital and second makes home visits (doctor, nurse, psychologist and a medical resident).	Hospitals readmissions over one year (type, number of times, days to readmission, and length of stay)<br><br>Quality of life (Sickness impact profile)	High risk of bias

For peer review only - http://bmjopen.bmj.com/site/about/guidelines.xhtml

						Telephone line available for careers			
Harris et al	2005	RCT	285	>55	Hospital at home	Seven days per week/10 hour per day nursing availability. 24 hour on-call geriatrician. Patient-centred planning. Daily nursing review and adjustment of individual care plan. Intensive home support with up to 24 hours support from a live-in home care professional. Multidisciplinary team support. Rehabilitation in the patient's home. A discharge hand-over to ongoing support services.	Nursing-led. Occupational therapist, physiotherapist, and social workers	Readmissions to hospital (first readmission in days 1-10,11-30 and 31-90)<br><br>Physical and mental function (functional independence)<br>Instrumental activities of daily living<br>Mental status<br>Self-reported recovery<br>Health status (SF-36)<br>Falls<br>Satisfaction with intervention<br><br>Costs	Low risk of bias
Hendrix et al	2013	Prospective controlled pre-post design	47	>60	Transition care	Hospital visit before the patient's discharge to initiate a relationship with the patient and begin planning for home care. Home visits by team. Educational materials for caregivers and information on community-based resources.	Nurse practitioner, social worker, occupational therapist	Emergency department visits and rehospitalisation at 30 and 60 days after hospital discharge.	Low quality and high risk of bias
Leung et al	2015	Matched-control quasi-experimental study	78	T: 80.2±7.7<br>C: 80.5±8.5	Hospital at home	The virtual ward physician might conduct a physician home visit within the first week after the patient was discharged. Based on the health assessment results, the virtual ward nurses planned the schedule of home visits, which could range from daily to once every 2 weeks.	Nurses and physicians	Unplanned emergency hospital readmissions, quality of life, emergency attendances	Low quality and unclear risk of bias
Low et al	2015	Prospective controlled pre-post design	259	>65	Transition care	An individualised patient-centred care plan drawn up for each patient after an initial home visit assessment. The frequency and timing of future home visits and the need to involve other members of the multi-disciplinary team is identified during the initial assessment and communicated to all team members. Nurses	Multi-disciplinary team comprising of a family physician, a nurse case manager, a physiotherapist, an occupational therapist, a speech therapist and a medical social worker. Nurses function as case managers.	Emergency department attendances (after 3 and 6 months)<br>Admissions to all hospitals (after 3 and 6 months)<br><br>Length of stay (after 3 and 6 months)<br><br>Specialist outpatient clinic	Low quality and high risk of bias

For peer review only - http://bmjopen.bmj.com/site/about/guidelines.xhtml

						make telephone call reviews based on patients' care needs and are accessible to patients by phone during office hours.		visits (after 3 and 6 months)	
Meret-Hanke	2011	Prospective controlled pre-post design	6992	>65	Case-management	PACE: Case-management services. Services provided in Day Health Centre (i.e.: recreational therapy nursing; Social services, including caregiver training, support groups, and respite care services) Dentistry). Patients attend centres from rarely to 7 days a week, depending on their care plans.	Comprehensive range of health care professionals	Hospital Use (as average days per month in hospital) (each 6 months for up to two years)	High risk of bias
Ong et al	2017	Retrospective non-controlled observational	107	>51.9	Case-management	Home-based medication reviews	Pharmacist and care coordinator	Hospital admissions<br>ED visits<br>Length of stay (days)	High risk of bias
Robinson et al	2015	Retrospective controlled pre-post design	5239	>65	Transition care	Transition intervention: post-discharge component (telephone assessment, education, support by team) to identify problems not dealt with during discharge, assist with self-management, and ensure appropriate health and social support	A geriatrician, a pharmacist and cultural support workers	28 day readmission rates<br>ED visits	Low risk of bias
Rytter el al	2010	RCT	331	>78	Transition care	Home visit by a GP and a district nurse one week after discharge, followed by two contacts after three and eight weeks	GP and nurse	Readmission rate within 26 weeks after discharge<br><br>Control of medication, evaluated 12 weeks after discharge	Low risk of bias
Sahota et al	2017	RCT and economic study	250	>70	Transition care	Community In-Reach rehabilitation and Care Transition (CIRACT). A comprehensive assessment of the patient's ability to perform different tasks and creation of a rehabilitation plan. Form links with the appropriate community services to ensure a smooth and effective discharge. Following discharge, team home visits to assess the level of rehabilitation	A senior occupational therapist (transition coach), a senior physiotherapist and an assistant practitioner, linked directly to a social services practitioner, working across multiple boundaries with patients and their carers.	Hospital length of stay<br><br>Readmission (28 and 91 days post-discharge)<br><br>Total time in bed days<br><br>Functional ability<br>Co-morbidity and health-related quality of life<br><br>Costs	Unclear risk of bias

For peer review only - http://bmjopen.bmj.com/site/about/guidelines.xhtml

						required. Further follow-up visits as deemed necessary and appropriate referral to additional community services.			
Stranges et al	2015	Retrospective cohort study	1144	>60	Transition care	Phone call 2 to 4 days after discharge. The patient seen in clinic, ideally within 1 week of discharge, by a social worker and a health professional. Home visit by health professional, who conducts a modified geriatric assessment with a focus on the reason for hospital admission.	Geriatric physicians, nurse practitioners, clinical pharmacists and social workers	30-day readmission rates	Low risk of bias
Young et al	2005	Prospective controlled pre-post design	1648	>63	Transition care	An intermediate care (IC) service. Joint care management team (multi-disciplinary, multi-agency) assesses need and purchases services for individuals delivered through a primary care trust. Patients accepted for IC are then assessed by each discipline in the team and a care plan is developed to be delivered by the care assistants. Patients receive input for up to 6 weeks, according to need.	Nurses (5), occupational therapists (2), care assistants (15), a dietician (half time) and access to psychiatric nurses	Nottingham Extended Activities of Daily Living score<br>Barthel Index<br>Hospital Anxiety and Depression score<br><br>Mortality (3, 6 and 12 months)<br>Readmission to hospital (3, 6 and 12 months)<br>Readmission to hospital number of days (3, 6 and 12 months)<br>New institutional care placements (3, 6 and 12 months)	Unclear risk of bias

For peer review only - http://bmjopen.bmj.com/site/about/guidelines.xhtml

Risk of bias

Two RCT studies were considered as presenting low risk of bias [20,26], while the others presented an unclear risk of bias due selective reporting that was discordant with the protocol [25], due to implementation problems and power analysis [18] and due to the randomisation process [15]. Two quasi-experimental studies presented low risk of bias [17,21], while the others were assessed as showing unclear risks due to a lack of randomisation [15,24,27] and programme implementation issues [21]. The remaining studies presented a high risk of bias due to elements of the methodological design or analysis [16,19,22,23,28] and baseline differences [14,27]. The assessment of risk of bias of individual studies is detailed in Appendix 4.

1) Types of Interventions

a) Transitional care

Transitional care is defined as a set of actions to ensure the coordination and continuity of health care as patients transfer between different locations; from hospital to nursing homes or between different levels of care within the same location [29]. Eight studies looked at transitional care interventions as a primary focus. Low et al [23] present a hospital-based intervention where patients received a comprehensive needs assessment in the home, followed by an individualised care plan that included medical and nursing care, patient education and coordination of care with other clinical specialists and community services. Nursing care included visits and follow-up phone calls. Robinson et al [21] describe an intervention that included an improved discharge process with two components: the identification of high risk patients and a post-discharge component that consisted of a nurse telephone assessment, along with education, and support by community nurses on the first and third days post-discharge. Sahota et al [18] present a programme with a pre-discharge functionality assessment tool and a prospective rehabilitation plan. After discharge, the service team performed home visits to assess progress and to provide appropriate referral to additional community services. Young et al [19] report on a city-wide intermediate care service where a joint care management team assessed patient needs and purchased support and rehabilitation from specialised teams.

Courtney et al [15] present an intervention portraying a comprehensive patient assessment and individualised care plan developed in consultation with the patient, health professionals, family, and caregivers. The intervention also had a weekly follow-up telephone call after discharge. Hendrix et al [16] present an intervention led by a nurse practitioner providing post-discharge medical care to patients for a time-limited period, with the addition of an occupational therapist and a social worker, who conducted separate home visits. Rytter et al [26] describe a joint home visit involving a GP and district nurse conducted one week after discharge and two contacts with the GP in the third and eighth weeks after discharge. Finally, Stranges et al [17] describe a multidisciplinary team of geriatric physicians, nurse practitioners, clinical pharmacists, and a social worker who assisted patients transitioning to the community. The intervention began with a pharmacist’s phone call 2 to 4 days after discharge, followed by a clinic appointment with a social worker and geriatrician or nurse within a week of discharge.

In summary, transitional care focused on the post-discharge component and covered a range of different actions such as the elaboration of individualised patient-centred care plans [15,23], telephone assessment and education [21] and home visits for short periods of time [17–19,23]. These

For peer review only - http://bmjopen.bmj.com/site/about/guidelines.xhtml

could lead to referrals to other services such as rehabilitation or additional community services [18,19]. Elements of transitional care are also apparent in some of the following intervention types.

b) Case-management services

Case management is a targeted, community-based, proactive approach to care that involves case-finding, assessment, care planning and care co-ordination [30]. Three studies had case-management services as their focus. Caplan et al [25] provide information on a comprehensive geriatric assessment performed in an emergency department, in addition to follow up visits by a hospital-based multidisciplinary outreach team over four weeks. Meret-Hanke [14] describes comprehensive medical and social services to targeted community-dwelling older individuals. The programme organised their services in a Day Health Centre which members attended with varying frequency (from occasionally to daily), depending on their care plans. De Stampa et al [24] examine a programme consisting of a physician and a case manager who conducted a home-based comprehensive geriatric assessment and produced individualised care plans using evidence-based interdisciplinary protocols. Finally, Ong et al present a home-based medication review programme led by a pharmacist, accompanied by a care coordinator, in liaison with other medical specialists. The programme provided pharmacological counselling, and the identification of drug-related problems [22].

All case-management interventions shared some characteristics: they included geriatric assessments, the elaboration of individualised care plans and coordination between care managers and/or multidisciplinary meetings where further interventions or referrals could be made [24,25]. While transitional care was not a focal point of this intervention type, the function of case management (as defined) was clearly concerned with ensuring a smooth transition between different services using various tools.

c) Hospital at home interventions

Hospital at home encompasses the active treatment at home by care professionals of people who would otherwise be admitted to hospital, and early supported discharge from hospital [24]. Three studies present information regarding hospital at home interventions. Espinel et al [27] describe a programme with visits by a multidisciplinary team, the provision of educational sessions and a telephone line available to carers, in addition to the provision of medical equipment and medication to the patients. Harris et al [20] describe an intervention that supported 10 hours per day of nursing, 24-hour on-call geriatricians, daily patient-centred planning by nurses and, if needed, the upgrading of individual care plans to offer intensive home support with up to 24-hours of a live-in home care professional. Finally, Leung et al [28] present a virtual ward service, whereby a nurse and a GP separately visit the home in the first week after discharge. The nurse visited the home every two weeks, providing psychosocial support to both patients and their carers. Additionally, a hotline consultation service was put in place. There are clear links to transitional care within this category.

2) Composition of Teams

A wide variety of multidisciplinary teams, in terms of composition and size, was observed across the studies. Two interventions were pharmacist-led, accompanied by other staff such as care coordinators [22] or cultural workers in a context of diversity [21]. All other studies described nurses playing leading roles, such as case managers [20,23,24]. Geriatricians were present in only one study [25] while family or primary care doctors were part of the intervention in three studies [23,24,27]. Other health workers

For peer review only - http://bmjopen.bmj.com/site/about/guidelines.xhtml

such as physiotherapists, occupational therapists, psychologists, speech therapists and dieticians were found in all studies [14,18,19,23–25,27]. Only three interventions included social workers [14,18,20].

3) Intervention effectiveness

a) Effects on hospital admissions and readmissions

Only the results of three studies assessed as presenting low risk of bias are presented. Only one intervention categorised as transitional care [17] found a significant reduction in 30 day readmission rates among matched compliers (n = 217, 11.7% vs 17.3%, treatment and control respectively; $p < .001$) and also a positive delay in terms of time to readmission, which was significantly longer among those receiving the intervention (18 ± 9 days compared with 12 ± 9 days with usual care; $p = .015$). Contrarily, neither Harris et al [20] (n=208, 76.8% vs 69.2%, treatment and control respectively; $p > .05$) nor Robinson et al [21] (n=2486, Regression discontinuity parameter estimation= -1.60 [SD 3.10]; $p > .05$) found statistically significant differences in terms of readmissions.

b) Healthcare expenditure

Harris et al report the costs of the hospital at home (NZ$6524) intervention were almost double standard hospital care (NZ$3525) [20], while Sahota et al found a net monetary benefit of £1,932 (95% CI= £2,134 to £5,863), although this has to be interpreted with caution given the wide confidence intervals [18]. Additionally, Courtey et al's intervention estimated an average cost saving of $333 for 24 weeks period per patient, and a net-monetary-benefit per individual of $7,907 [31].

4) Type of outcomes

Ten studies reported on different lengths of time of hospital readmissions [16–21,26–28], while six reported hospital admissions [14,18,22–25]. Only one study presented both outcomes [18]. Six studies reported emergency department admissions [15,21–25] and five measured different lengths of hospital stay [14,18,22,23,27]. Additionally, three studies measured emergency GP visits [15,16,28], and one also measured control of medication [26].

Other outcomes, such as functional status and quality of life or depression, were measured in several prospective studies [18–20,24,27,28]. Mortality was assessed in two studies [19,25] and there was a costs analysis in three studies [15,18,20], one of them in a separate study [31]. Finally, patient satisfaction is assessed in one study [20].

Results were assessed over a wide range of time periods. One study started measuring results 10 days after the intervention started [20], while the most common follow-up period ranged from 1 to 6 months [14–21,23,25,26,28]. Two studies extended the follow up period to one year [19,27] and one measured the effects after two years [14].

DISCUSSION

Hypothesis traps limit relevant results

Different concurrent explanations may explain the limited number of studies that produce significant statistical differences. One plausible explanation, known as the 'omitted variable bias', notes that outcome measures such as hospital readmissions and length of stay are moderated by other confounding variables that are usually not captured in large-sample studies. Conversely, the same

For peer review only - http://bmjopen.bmj.com/site/about/guidelines.xhtml

outcomes cannot be easily observed in smaller-sample in-depth studies, due to the impact of sample size in hypothesis testing.

Another explanation is the 'outcome selection trap', which refers to the practice of identifying relevant and easy-to-collect outcomes (therefore generating less measurement error) in intervention studies. However, those outcomes could be considered excessively distant from the nature of the interventions. For instance, valuable low-impact interventions such as follow-up phone calls should not be assessed in terms of reduction of hospitalisation, but in terms of other subjective measures such as quality of life or process-related outputs such as correct use of medication.

A third explanation is the 'causality gap', which refers to the inability of many of those interventions to establish a clear theoretical causal connection between, on one hand, activities and resources and, on the other, outputs and outcomes. This includes an inability to estimate the proportion of resources, time and activities needed to achieve specific measurable changes, such improvements in health status.

A fourth explanation is the 'timeframe gap', whereby studies do not analyse effects over a sufficiently long period to observe changes that accrue over lengthier time frames.

A fifth explanation is the 'instrument gap', which refers to the limitations of chiefly using outcomes such as hospital admissions, readmissions or length of stay to measure interventions and not developing adequate tools to identify and measure frailty or including the older person's perspective.

The diversity and limitations of interventions

The review included different interventions focusing on reducing the avoidable displacement from home of older people, varying from a range of complex designs such as hospital at home programmes to simpler ones such as medication reviews. These interventions occurred over different lengths of time, including short-term transitional care services and longer ones such as hospital at home assistance and case-management interventions. The number of caregivers, the permanent, periodical or sporadic nature of their work, and their specialisations varied significantly.

All fifteen studied interventions were based almost entirely within the health system and were provided by a wide range of health specialists. With the exception of three studies [16–18], there was no integration between mainstream health services and other agencies, such as housing services or social care.

At the same time, there was an absence of preventive interventions. All the quasi-experiments and RCT review interventions viewed the hospital setting as the 'initial status' of the intervention. Only one study emphasised preventive care through regular clinical monitoring [25]. This is especially relevant for interventions that focus on a wide range of older people with different health statuses and different degrees of functional dependency.

With respect to the intervention types (transitional care, case management and hospital at home), the overlaps were unsurprising, given their definitions and the complex arrangement of services and actions needed to care for frail older people within an integrated care context. Some parallels with other broad-based initiatives such as intermediate care can be seen. In the UK where intermediate care concepts have been operationalised, they include four types of intervention: crisis response,

For peer review only - http://bmjopen.bmj.com/site/about/guidelines.xhtml

home-based care, bed-based care and reablement [32]. The category of home-based care in this review corresponds with the hospital at home category.

Assessing interventions with the 'avoidable displacement from home' framework

The avoidable displacement from home framework appears to be a useful lens through which to assess integrated care interventions. That said, the three key values of ADH (person-centredness, place-based care and sustainability) did not emerge prominently in this review. None of the interventions reviewed referred to all of these elements. Two management service interventions [24,25] might be characterised by a person-centred approach with a focus on individual rehabilitation and care plans. However, neither study specifically assessed these interventions from a person-centred perspective. This was an important omission which might have been averted through more robust application of the ADH framework. Likewise, including place-based care as an analytical theme would have revealed degrees of service fragmentation, especially for interventions that seek to integrate external care with services at home [32]. It was not possible to determine the degree of service integration for most interventions, but the available evidence for the medication review intervention indicated that it did not fully satisfy Kodner and Kyriacou's definition of integrated care [8]. In terms of intervention sustainability, many studies focused on short-term interventions and outcomes (1 to 6 months), which was not necessarily compatible with meeting the chronic, complex needs associated with older age. In terms of economic sustainability, only three studies provided any element of economic analysis [18,20,31], reflecting the numerous challenges of quantifying cost benefits for integrated care interventions [33].

Comparison with previous literature reviews

Four previous systematic reviews [34–37] examined the effectiveness of different types of interventions to prevent hospitalisations based on home care and demonstrated the limitations of current impact evidence, in terms of consistent positive outcomes. Huntley et al [38] reviewed evidence about alternatives to acute care, such as paramedic/emergency department-based interventions and community hospitals. They reported that impact evidence was limited by a lack of systematic data on outcomes and costs for patients, health professionals or carers. In a review of integrated care interventions for older people, Baxter et al found inconsistent effects on admissions, length of stay and costs [39].

Limitations of this review

This review looks at interventions to reduce avoidable displacement from home that are conducted in home settings. It does not consider other settings, including those that may be applied in hospitals and nursing homes, which should be the focus of future reviews.

All the articles but one in this review studied health care systems in high-income countries, even though low or middle-income countries contain a larger number of older people with health and care needs. This may reflect the limited number of languages included in the study, although that is unlikely to have excluded a large number of studies.

Empirical evidence in this area is predominately from quasi-experiments and observational studies, while studies assessed as having a low risk of bias were an exception.

For peer review only - http://bmjopen.bmj.com/site/about/guidelines.xhtml

Although the search strategy focused on hospital avoidance, all included interventions were related to admissions and readmissions. Other search strategies could inform on interventions promoting hospital admission avoidance.

This review is limited to quantitative findings due to the heterogeneity of the qualitative studies in terms of frame of reference and focus of enquiry. Future reviews should seek to address this challenge in order to maximise the available evidence.

CONCLUSIONS AND IMPLICATIONS

One purpose of this review was to establish the potential validity of the concept of avoidable displacement from home. The review was not able to identify robust impact evidence either in terms of quantity or quality from the studies presented. Therefore, the evidence cannot be considered sufficiently strong to inform policy or interventions seeking to reduce avoidable displacement from home. The paucity of evidence does not result from the limited number of potentially relevant interventions. Rather, it reflects the complexity of these interventions and a lack of systematic data collection. As such, this review identifies an urgent need for systematic monitoring and data management, as well as enhanced data collection. Data should be derived from a more careful selection of measurement instruments, implementation strategies and robust methods for evaluating multifaceted interventions in complex populations.

Contributors: LS: lead systematic reviewer conducting all stages of the review and was responsible for the initial draft of protocol and draft of paper. JB: second reviewer; contributing to discussion as the review progressed; commenting and editing on the drafts of the paper. PLS: secured funding, idea of current work, third reviewer and contributing to discussion as the review progressed; commenting and editing on the drafts of the paper.

Acknowledgements: The authors thank the invaluable contribution of the reviewers: Gemma Hughes, Asangaedem Akpan and Ken Hillman as well of BMJ Open editor.

Funding: This paper presents research funded by Eastern ARC Consortium and Medical Research Council (grant reference number MR/R024219/1).

Disclaimer: The views expressed are those of the authors and not necessarily those of the Eastern ARC Consortium and Medical Research Council

Competing interests: None declared.

Patient consent for publication: Not required

Provenance and peer review: Not commissioned; externally peer reviewed.

Data sharing statement: No additional data are available.

Open access: This is an open access article distributed in accordance with the Creative Commons Attribution 4.0 Unported (CC BY 4.0) license, which permits others to copy, redistribute, remix, transform and build upon this work for any purpose, provided the original work is properly cited, a link to the licence is given, and indication of whether changes were made. See: https://creativecommons.org/ licenses/by/4.0/.

For peer review only - http://bmjopen.bmj.com/site/about/guidelines.xhtml

REFERENCES

1 United Nations. Department of Economic and Social Affairs. Population Division. World Population Ageig 2017 - Highlights (ST/ESA/SER.A/397). 2017. doi:10.1049/el:20000788

2 Clegg A, Young J, Iliffe S, *et al.* Frailty in elderly people. *Lancet* 2013;**381**:752–62. doi:10.1016/S0140-6736(12)62167-9

3 British Geriatric Society. Fit for Frailty - consensus best practice guidance for the care of older people living in community and outpatient settings -. 2014.

4 Souza DK de, Peixoto SV. Estudo descritivo da evolução dos gastos com internações hospitalares por condições sensíveis à atenção primária no Brasil, 2000-2013. *Epidemiol e Serviços Saúde* 2017;**26**:285–94. doi:10.5123/s1679-49742017000200006

5 Wright PN, Tan G, Iliffe S, *et al.* The impact of a new emergency admission avoidance system for older people on length of stay and same-day discharges. *Age Ageing* 2013;**43**:116–21. doi:10.1093/ageing/aft086

6 Charles A, Wenzel L, Kershaw M, *et al.* A year of integrated care systems: reviewing the journey so far. 2018.

7 Kodner DL, Kyriacou CK. Fully integrated care for frail elderly: two American models. *Int J Integr Care* 2000;**1**. doi:10.5334/ijic.11

8 Briggs A, Araujo de Carvalho I. Actions required to implement integrated care for older people in the community using the World Health Organization ' s ICOPE approach : A global Delphi consensus study. *PLoS One* 2018;**13**:1–16.

9 Lloyd-Sherlock P, Billings J, Giacomin KC, *et al.* Meeting the complex challenge of health and social care provision for rapidly-ageing populations. Introducing the concept of "avoidable displacement from home". Manuscr. Submitt. Publ. 2019.

10 McCormack B. Person-centredness in gerontological nursing: an overview of the literature. *J Clin Nurs* 2004;**13**:31–8. doi:10.1016/j.maturitas.2016.07.010

11 Ham CAH. Place-based Health Systems. *King's Fund* 2015;:1–50.http://www.kingsfund.org.uk/sites/files/kf/field/field_publication_file/Place-based-systems-of-care-Kings-Fund-Nov-2015_0.pdf

12 de Bruin SR, Stoop A, Billings J, *et al.* The SUSTAIN Project: A European Study on Improving Integrated Care for Older People Living at Home. *Int J Integr Care* 2018;**18**:1–12. doi:10.5334/ijic.3090

13 Higgins JP, Green S. *Cochrane Handbook for Systematic Reviews of Interventions*. 2011. doi:10.1002/9780470712184.ch5

14 Meret-Hanke LA. Effects of the program of all-inclusive care for the elderly on hospital use. *Gerontologist* 2011;**51**:774–85. doi:10.1093/geront/gnr040

15 Courtney M, Edwards H, Chang A, *et al.* Fewer emergency readmissions and better quality of life for older adults at risk of hospital readmission: A randomized controlled trial to determine the effectiveness of a 24-week exercise and telephone follow-up program. *J Am Geriatr Soc*

For peer review only - http://bmjopen.bmj.com/site/about/guidelines.xhtml

2009;**57**:395–402. doi:10.1111/j.1532-5415.2009.02138.x

16 Hendrix C, Tepfer S, Forest S, *et al.* Transitional Care Partners: a hospital-to-home support for older adults and their caregivers. *J Am Assoc Nurse Pract* 2013;**25**:407–14. doi:10.1111/j.1745-7599.2012.00803.x

17 Stranges PM, Marshall VD, Walker PC, *et al.* A Multidisciplinary Intervention for Reducing Readmissions Among Older Adults in a Patient-Centered Medical Home. *Clinical* 2015;**21**:106–13.

18 Sahota O, Pulikottil-Jacob R, Marshall F, *et al.* The Community In-reach Rehabilitation and Care Transition (CIRACT) clinical and cost-effectiveness randomisation controlled trial in older people admitted to hospital as an acute medical emergency. *Age Ageing* 2017;**46**:26–32. doi:10.1093/ageing/afw149

19 Young JB, Robinson M, Chell S, *et al.* A whole system study of intermediate care services for older people. *Age Ageing* 2005;**34**:577–83. doi:10.1093/ageing/afi179

20 Harris R, Ashton T, Broad J, *et al.* The effectiveness, acceptability and costs of a hospital-at-home service compared with acute hospital care: a randomized controlled trial. *J Heal Serv Res Policy* 2005;**10**:158–66.http://onlinelibrary.wiley.com/o/cochrane/cleed/articles/NHSEED-22005007671/frame.html

21 Robinson TE, Zhou L, Kerse N, *et al.* Evaluation of a New Zealand program to improve transition of care for older high risk adults. *Australas J Ageing* 2015;**34**:269–74. doi:10.1111/ajag.12232

22 Ong KY, Cheen MHH, Chng JSG, *et al.* Effectiveness of a multidisciplinary home-based medication review program in reducing healthcare utilization among older adult Singaporeans. *Geriatr Gerontol Int* 2017;**17**:302–7. doi:10.1111/ggi.12706

23 Low LL, Vasanwala FF, Ng LB, *et al.* Effectiveness of a transitional home care program in reducing acute hospital utilization: A quasi-experimental study. *BMC Health Serv Res* 2015;**15**:1–8. doi:10.1186/s12913-015-0750-2

24 de Stampa M, Vedel I, Buyck J-F, *et al.* Impact on hospital admissions of an integrated primary care model for very frail elderly patients. *Arch Gerontol Geriatr* 2014;**58**:350–5. doi:10.1016/J.ARCHGER.2014.01.005

25 Caplan GA, Williams AJ, Daly B, *et al.* A Randomized , Controlled Trial of Comprehensive Geriatric Elderly from the Emergency Department - The DEED II Study. *J Am Geriatr Soc* 2004;**52**:1417–23.

26 Rytter L, Jakobsen HN, Rønholt F, *et al.* Comprehensive discharge follow-up in patients' homes by GPs and district nurses of elderly patients. *Scand J Prim Health Care* 2010;**28**:146–53. doi:10.3109/02813431003764466

27 Espinel-Bermúdez MC, Sánchez-García S, Juárez-Cedillo T, *et al.* Impacto de un programa de atención domiciliaria al enfermo crónico en ancianos: Calidad de vida y reingresos hospitalarios. *Salud Publica Mex* 2011;**53**:17–25. doi:10.1590/S0036-36342011000100004

28 Leung DYP, Lee DTF, Lee IFK, *et al.* The effect of a virtual ward program on emergency

For peer review only - http://bmjopen.bmj.com/site/about/guidelines.xhtml

services utilization and quality of life in frail elderly patients after discharge: A pilot study. *Clin Interv Aging* 2015;**10**:413–20. doi:10.2147/CIA.S68937

29 Coleman E, Boult C. Improving the Quality of Transitional Care for Persons with Complex Care Needs. *J Am Geriatr Soc Am Geriatr Soc* 2003;**51**:556–7.

30 Ross S, Curry N, Goodwin N. Case management: What it is and how it can best be implemented. 2011.

31 Graves N, Courtney M, Edwards H, *et al.* Cost-Effectiveness of an Intervention to Reduce Emergency Re-Admissions to Hospital among Older Patients. *PLoS One* 2009;**4**:e7455. doi:10.1371/journal.pone.0007455

32 Winfield A, Burns E. Let’s all get home safely: a commentary on NICE and SCIE guidelines (NG27) transition between inpatient hospital settings and community or care home settings. *Age Ageing* 2016;**45**:757–60. doi:10.1093/ageing/afw151

33 Evers S, Paulus AT. Health Economics and Integrated Care: a growing and challenging relationship. *Int J Integr Care* 2015;**15**:1–2. doi:10.5334/ijic.2201

34 Shepperd S, Iliffe S, Doll H, *et al.* Admission avoidance hospital at home. *Cochrane Database Syst Rev* 2016;**2016**. doi:10.1002/14651858.CD007491.pub2

35 Oeseburg B, Wynia K, Middel B, *et al.* Effects of Case Management for Frail Older People or Those With Chronic Illness. A Systematic Review. *Nurs Res* 2009;**58**:201–10.

36 Linertová R, García-Pérez L, Vázquez-Díaz JR, *et al.* Interventions to reduce hospital readmissions in the elderly: In-hospital or home care. A systematic review. *J Eval Clin Pract* 2011;**17**:1167–75. doi:10.1111/j.1365-2753.2010.01493.x

37 Huntley AL, Thomas R, Mann M, *et al.* Is case management effective in reducing the risk of unplanned hospital admissions for older people? A systematic review and meta-analysis. *Fam Pract* 2013;**30**:266–75. doi:10.1093/fampra/cms081

38 Huntley AL, Chalder M, Shaw ARG, *et al.* A systematic review to identify and assess the effectiveness of alternatives for people over the age of 65 who are at risk of potentially avoidable hospital admission. *BMJ Open* 2017;**7**:1–7. doi:10.1136/bmjopen-2017-016236

39 Baxter S, Johnson M, Chambers D, *et al.* The effects of integrated care: A systematic review of UK and international evidence. *BMC Health Serv Res* 2018;**18**:1–13. doi:10.1186/s12913-018-3161-3

For peer review only

For peer review only - http://bmjopen.bmj.com/site/about/guidelines.xhtml

Legends:

Figure 1. PRISMA flow diagram of literature review

For peer review only

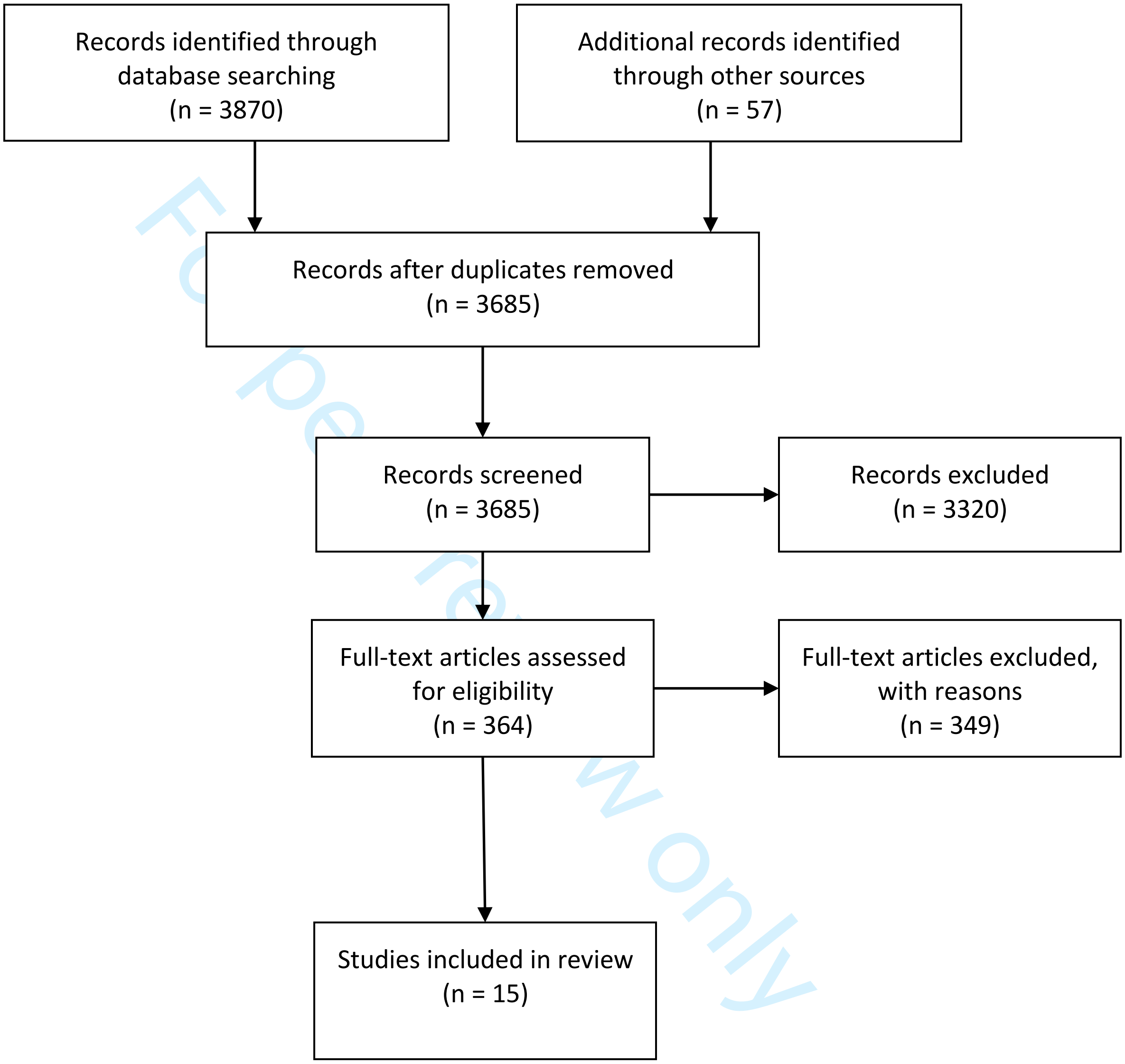

From: Moher D, Liberati A, Tetzlaff J, Altman DG, The PRISMA Group (2009). *P*referred *R*eporting *I*tems for *S*ystematic Reviews and *M*eta-*A*nalyses: The PRISMA Statement. PLoS Med 6(7): e1000097. doi:10.1371/journal.pmed1000097

For more information, visit www.prisma-statement.org.

For peer review only - http://bmjopen.bmj.com/site/about/guidelines.xhtml

APPENDIX 1: SEARCH STRATEGY

"home" OR "home hospital" OR "out-patient facility" OR "hospital in home" OR "Home care" OR "Domiciliary care" OR "outreach programme" OR "hospital outreach" OR "comprehensive elderly care" OR "home hospitalisation" AND "Frail elderly" OR "Elderly, Frail" OR "Functionally-impaired Elderly" OR "Aged OR elderly" OR "Aged, 80 and over" OR "Centenarians" OR "Nonagenarians" OR "Octogenarians" AND "Involuntary hospitalisation" OR "Avoidable hospitalisation" OR "Patient admission" OR "Voluntary Admission" OR "Patient Readmission" OR "30 Day Readmission" OR "Thirty Day Readmission" OR "Hospital Readmissions" OR "Readmissions, Hospital".

APPENDIX 2: DATA EXTRACTION FORM

Title
Author
Year
Methods
Participants
Patients
Classification of intervention
Intervention
Team
Person centeredness
Sustainability
Preventive aspect
Outcomes
Results
Conclusion

For peer review only

APPENDIX 3: RISK BIAS

Author	Random sequence generation (selection bias)	Allocation concealment (selection bias)	Blinding of participant and personnel (performance bias)	Blinding of outcome assessment (detection bias)	Incomplete outcome data (attrition bias)	Selective reporting bias
Caplan et al	Unclear risk (block randomisation)	Low risk (opaque envelopes)	Higk risk ("almost half conducted by a research assistant, with the rest performed by different members of the multidisciplinary team")	Low risk. Baseline characteristics are similar	"Unclear (10.0% of intervention participants and 13.0% of control participants withdrew from the study. The authors did not report reasons for withdrawal.)"	"High risk (Only data at 1 and 18 months were reported, yet measurements were taken at 1, 3, 6, 12 and 18 months)"
Courtney et al	Low risk (computer-generated)	High risk (not mentioned)	Unclear risk (not mentioned)	Low risk. Baseline characteristics are similar	High risk (attrition and 28% lack of compliance)	Low risk. Main outcomes are reported
de Stampa el al	High risk. 'Patients were recruited consecutively until the desired sample sizes were achieved.'		Low risk. Interventions occurs in different geographical areas	Low risk. Similar baseline characteristics	Low risk. Attrition is similar in both groups (deceased or institutionalised)	Low risk. Results are reported as stated in protocol
Espinel-Bermudez et al	High risk. Unclear criteria of selection (mentions quota and convenience sampling)		Low risk. (Two different hospitals, doing ordinary activities)	High risk. Baseline 'Length of stay' and comorbidity index differs between groups.	High risk. Unbalanced groups leads to different attrition loss.	Unclear
Harris et al	Low risk (computer-generated)	Unclear. Do not mention allocation process	Low risk (“Interviews by trained researchers who were not involved in patient care, but who were	Low risk (baseline results similar)	Unclear. Loss to follow-up: hospital at home: 14/143; hospital: 17/142	High risk (no mention to registered protocol)

			not blinded as to the intervention group")			
**Hendrix et al**	DNA	DNA	DNA	DNA	Low risk	High risk (absence of statistical analysis)
**Leung et al**	DNA	DNA	DNA	DNA	Low risk (no loss of patients)	High risk (statistical analysis incomplete)
**Low et al**	DNA	DNA	DNA	DNA	Low risk. Loss of 3 out of 262 patients	High risk. Does not control for covariables
**Meret-Hanke**	Low risk. "First, propensity score matching assumes that outcomes are independent of treatment assignment given an observable set of covariates (Dehejia & Wahba, 2002; Rosenbaum & Rubin, 1983). Second, the propensity score distributions of the treatment and comparison groups are assumed to overlap (Rosenbaum & Rubin, 1983). To satisfy these assumptions, we included covariates that strongly affect both the treatment decision and the outcome to effectively reduce bias (Smith & Todd, 2005)."		Low risk. Retrospective analysis	High risk. "For instance, individuals who enroll in PACE could be more averse to using hospitals than other individuals." "In addition, although we were able to match on most individual characteristics, we were unable to match on market, geographic, and historical factors."	Low risk. Different attrition bias analysis performed (baseline characteristics lost to attrition, effect of cases on outcomes)	Unclear

**Ong et al**	DNA	DNA	DNA	DNA	Low risk. 12% of deaths	Low risk. Main outcomes are reported
**Robinson et al**	Low risk. Threshold for RD is the minimum required for treatment ('risks core where the patients begin to receive the intervention(20% risk)')		Low risk. Quasi-experimental design (RD and ITS)	Low risk. Quasi-experimental design (RD and ITS)	Low risk. 'From this target group we identified eligible 'High Risk' patients using a predictive risk model (PRM) that could be run early in the patient's admission'	Low risk ("We measured whether the intervention was delivered according to the initial plan. In particular we measured whether patients received the discharge pharmacy and the nurse telephone follow-up services using program administrative records")
**Rytter et al**	Low risk (computer generated)	Low risk (opaque envelopes)	High risk (not blinded to GP and nurses)	Low risk (baseline characteristics similar)	Unclear risk (Dropouts of lower functional ability patients)	Low risk. Main outcomes reported.

For peer review only - http://bmjopen.bmj.com/site/about/guidelines.xhtml

**Sahota et al**	Low risk (computer-generated)	Low risk (The randomisations will be requested through a PC with internet explorer and internet access. The system is located on a dedicated secure server within the University of Nottingham)	Low risk. Protocol: The RA collecting data and the research team analysing the data will be blinded to treatment allocation. The participant and ward staff will not be blinded to treatment allocation as the treating therapists will be liaising closely with ward staff to ensure optimal patient care. The three-month follow-up data will be collected by an RA blinded to treatment allocation.	Low risk (The groups appeared well balanced at baseline)	Low risk (212 participant, 106 in each arm)	Low risk (similar outcomes as protocol)
**Stranges et al**	DNA	DNA	DNA	Low risk (Propensity Score Matching)	Low risk (retrospective analysis)	Low risk. Main outcomes are reported
**Young et al**	High risk. Patients were recruited consecutively		Unclear. Evaluation occurs within policy change	Low risk. Baseline characteristics are similar	Unclear. Mortality rate is similar after 1 year	Low risk. Main outcomes are reported

DNA: Do not apply

APPENDIX 4: PRISMA CHECKLIST

Section/topic	#	Checklist item	Reported on page #
TITLE			
Title	1	Identify the report as a systematic review, meta-analysis, or both.	1
ABSTRACT			
Structured summary	2	Provide a structured summary including, as applicable: background; objectives; data sources; study eligibility criteria, participants, and interventions; study appraisal and synthesis methods; results; limitations; conclusions and implications of key findings; systematic review registration number.	2
INTRODUCTION			
Rationale	3	Describe the rationale for the review in the context of what is already known.	3
Objectives	4	Provide an explicit statement of questions being addressed with reference to participants, interventions, comparisons, outcomes, and study design (PICOS).	4
METHODS			
Protocol and registration	5	Indicate if a review protocol exists, if and where it can be accessed (e.g., Web address), and, if available, provide registration information including registration number.	4
Eligibility criteria	6	Specify study characteristics (e.g., PICOS, length of follow-up) and report characteristics (e.g., years considered, language, publication status) used as criteria for eligibility, giving rationale.	4-5
Information sources	7	Describe all information sources (e.g., databases with dates of coverage, contact with study authors to identify additional studies) in the search and date last searched.	4
Search	8	Present full electronic search strategy for at least one database, including any limits used, such that it could be repeated.	4 AND APPENDIX
Study selection	9	State the process for selecting studies (i.e., screening, eligibility, included in systematic review, and, if applicable, included in the meta-analysis).	5
Data collection process	10	Describe method of data extraction from reports (e.g., piloted forms, independently, in duplicate) and any processes for obtaining and confirming data from investigators.	5
Data items	11	List and define all variables for which data were sought (e.g., PICOS, funding sources) and any assumptions and simplifications made.	4
Risk of bias in individual studies	12	Describe methods used for assessing risk of bias of individual studies (including specification of whether this was done at the study or outcome level), and how this information is to be used in any data synthesis.	5
Summary measures	13	State the principal summary measures (e.g., risk ratio, difference in means).	DNA
Synthesis of results	14	Describe the methods of handling data and combining results of studies, if done, including measures of consistency (e.g., I^2) for each meta-analysis.	DNA

For peer review only

Risk of bias across studies	15	Specify any assessment of risk of bias that may affect the cumulative evidence (e.g., publication bias, selective reporting within studies).	5 and APPENDIX
Additional analyses	16	Describe methods of additional analyses (e.g., sensitivity or subgroup analyses, meta-regression), if done, indicating which were pre-specified.	DNA
**RESULTS**			
Study selection	17	5-6	4
Study characteristics	18	For each study, present characteristics for which data were extracted (e.g., study size, PICOS, follow-up period) and provide the citations.	6-11
Risk of bias within studies	19	Present data on risk of bias of each study and, if available, any outcome level assessment (see item 12).	12 and APPENDIX
Results of individual studies	20	For all outcomes considered (benefits or harms), present, for each study: (a) simple summary data for each intervention group (b) effect estimates and confidence intervals, ideally with a forest plot.	12-14
Synthesis of results	21	Present results of each meta-analysis done, including confidence intervals and measures of consistency.	DNA
Risk of bias across studies	22	Present results of any assessment of risk of bias across studies (see Item 15).	DNA
Additional analysis	23	Give results of additional analyses, if done (e.g., sensitivity or subgroup analyses, meta-regression [see Item 16]).	DNA
**DISCUSSION**			
Summary of evidence	24	Summarize the main findings including the strength of evidence for each main outcome; consider their relevance to key groups (e.g., healthcare providers, users, and policy makers).	6-11
Limitations	25	Discuss limitations at study and outcome level (e.g., risk of bias), and at review-level (e.g., incomplete retrieval of identified research, reporting bias).	16
Conclusions	26	Provide a general interpretation of the results in the context of other evidence, and implications for future research.	15-17
**FUNDING**			
Funding	27	Describe sources of funding for the systematic review and other support (e.g., supply of data); role of funders for the systematic review.	17

From: Moher D, Liberati A, Tetzlaff J, Altman DG, The PRISMA Group (2009). Preferred Reporting Items for Systematic Reviews and Meta-Analyses: The PRISMA Statement. PLoS Med 6(7): e1000097. doi:10.1371/journal.pmed1000097

For peer review only
